# Relationship between Research Outcomes and Risk of Bias, Study Sponsorship, and Author Financial Conflicts of Interest in Reviews of the Effects of Artificially Sweetened Beverages on Weight Outcomes: A Systematic Review of Reviews

**DOI:** 10.1371/journal.pone.0162198

**Published:** 2016-09-08

**Authors:** Daniele Mandrioli, Cristin E Kearns, Lisa A. Bero

**Affiliations:** 1 Cesare Maltoni Cancer Research Center, Ramazzini Institute, Bentivoglio, Bologna, Italy; 2 Johns Hopkins Bloomberg School of Public Health, Baltimore, Maryland, United States of America; 3 Philip R. Lee Institute for Health Policy Studies, University of California San Francisco, San Francisco, California, United States of America; 4 Department of Orofacial Sciences, School of Dentistry, University of California San Francisco, San Francisco, California, United States of America; 5 Charles Perkins Centre and Faculty of Pharmacy, University of Sydney, Sydney, New South Wales, Australia; Royal College of Surgeons in Ireland, IRELAND

## Abstract

**Background:**

Artificially sweetened beverage consumption has steadily increased in the last 40 years. Several reviews examining the effects of artificially sweetened beverages on weight outcomes have discrepancies in their results and conclusions.

**Objectives:**

To determine whether risk of bias, results, and conclusions of reviews of effects of artificially sweetened beverage consumption on weight outcomes differ depending on review sponsorship and authors’ financial conflicts of interest.

**Methods:**

We performed a systematic review of reviews of the effects of artificially sweetened beverages on weight. Two assessors independently screened articles for inclusion, extracted data, and assessed risks of bias. We compared risk of bias, results and conclusions of reviews by different industry sponsors, authors’ financial conflict of interest and journal sponsor. We also report the concordance between review results and conclusions.

**Results:**

Artificial sweetener industry sponsored reviews were more likely to have favorable results (3/4) than non-industry sponsored reviews (1/23), RR: 17.25 (95% CI: 2.34 to 127.29), as well as favorable conclusions (4/4 vs. 15/23), RR: 1.52 (95% CI: 1.14 to 2.06). All reviews funded by competitor industries reported unfavorable conclusions (4/4). In 42% of the reviews (13/31), authors’ financial conflicts of interest were not disclosed. Reviews performed by authors that had a financial conflict of interest with the food industry (disclosed in the article or not) were more likely to have favorable conclusions (18/22) than reviews performed by authors without conflicts of interest (4/9), RR: 7.36 (95% CI: 1.15 to 47.22). Risk of bias was similar and high in most of the reviews.

**Conclusions:**

Review sponsorship and authors’ financial conflicts of interest introduced bias affecting the outcomes of reviews of artificially sweetened beverage effects on weight that could not be explained by other sources of bias.

## Introduction

The global obesity epidemic represents one of the biggest public health challenges of our time: there are currently 600 million adults and 42 million children that are obese [1]. Overweight and obesity are associated with increased risk for a variety of chronic and debilitating diseases including cancer, cardiovascular disease and diabetes [2]. Once considered a problem only in high-income countries, overweight and obesity are now dramatically on the rise in low- and middle-income countries. People, particularly women, with less education and lower socio-economic status are more likely to be obese [3].

While no single factor is responsible for the recent increases in overweight and obesity, excess calories and inadequate physical activity are known determinants [4]. Added sugars are a significant source of excess calories and sugar-sweetened beverages are one of the main sources of added sugars, with an estimated 184 000 annual deaths attributable to their consumption worldwide [5, 6]. Multiple agencies have recommended daily limits for added sugars [7–9], as well as limitations on sugar sweetened beverage consumption [10].

Evidence of the adverse effects of added sugars on weight gain and obesity has fueled a debate about whether they should be replaced by artificial sweeteners [11]. Artificially sweetened beverage consumption, considered the main source of artificial sweeteners in the diet, has dramatically increased in the last 40 years in children and adults [12–17]. However, the replacement of added sugars with artificial sweeteners to prevent and control obesity is controversial, due to safety concerns [18–20] and conflicting evidence on their effect on weight [7, 21, 22]. Some studies have shown that artificial sweeteners may negatively affect the gut microbiome and pathways associated with diabetes mellitus or obesity in both rodents and humans [23–25]. Reverse causality has been proposed as a possible explanation for the discrepancies in the evaluation of the effects on weight of artificially sweetened beverages, since individuals at higher risk for weight gain may choose to consume artificially sweetened beverages in an attempt to control their weight [26]. Some authors suggest that artificially sweetened beverage consumption could lead to caloric compensation [27], while others suggest that satiety levels do not differ between people consuming different sweeteners [28].

Another possible explanation for the conflicting results of studies of artificially sweetened beverages may be bias related to funding source. Industry sponsorship of both original research and review articles is associated with favorable outcomes for the sponsor in a variety of areas including clinical drug trials [29, 30], studies of the health effects of tobacco [31, 32], medical procedures [33, 34] and pre-clinical animal studies [35, 36]. The observed bias related to funding is not explained by other risks of bias in the studies (for example sequence generation, concealment of allocation, or loss to follow-up). In addition, bias may be related to funding source even when all studies are industry-funded. For example, among industry-sponsored head-to-head comparisons of statin drugs, the results favor the statin made by the company that sponsored the study [37].

Developers of public health guidelines have been adopting systematic review methods and more structured methods for grading recommendations; assessing bias in the reviews is a critical step in the process [7, 38, 39]. The food and beverage industry frequently sponsors research on the health effects of added sugars consumption and has produced reviews for policy purposes. For example, the cane and beet sugar industry has lobbied public health organizations, such as the World Health Organization, and produced reviews critical of the role of sugar in dental caries, overweight and obesity, and atherosclerotic vascular disease [40, 41]. Reviews of the health effects of sugar sweetened beverages that are performed by authors with financial ties to food companies are five times more likely to conclude that there is no association of sugar consumption with weight gain compared to those with other sponsors (relative risk: 5.0, 95% CI: 1.3–19.3)[42].

Low-calorie and caloric sweetener industries sponsor studies and reviews of the health effects of artificially sweetened beverages. There is some evidence of funding bias in original research studies. Walton has reported that among a sample of studies of aspartame, 100% of the industry sponsored studies concluded that aspartame was safe, and 92% of the independently funded studies identified adverse effects of aspartame consumption [43]. Millstone observed that in a recent re-evaluation of aspartame safety by the European Food Safety Authority, 97% of the studies that reported no harm were industry sponsored, while 100% of the studies indicating possible harms were non-industry sponsored [44]. The relationship of funding source and other biases to the outcomes of reviews of the effects of artificially sweetened beverages on weight outcomes has not been evaluated.

The objectives of this systematic review are to determine whether risk of bias, results, and conclusions of reviews of the effects of artificially sweetened beverage consumption on weight outcomes differ depending on 1) sources of review sponsorship and 2) authors’ financial conflicts of interest.

Our *a priori* hypothesis is that review sponsorship and authors’ financial conflicts of interest will be associated with results and conclusions that favor the sponsor’s product, and that risk of bias will not differ by sponsor or author financial conflicts of interest.

## Methods

The selection criteria for reviews, data extraction and analyses were determined *a priori* to data collection. This research was exempt from Institutional Review Board approval as all the data were publically available.

### Inclusion / Exclusion Criteria for Reviews

We included published reviews that included human research studies comparing one or more artificially sweetened beverages to water, sugar sweetened beverages or mixed comparisons where the effects on weight were evaluated as a primary or secondary outcomes through BMI score or other measures of overweight and obesity. Overweight is defined as BMI > 25 kg/m^2^ and obesity as BMI > 30 kg/m^2^.

An article was included if its stated or implied purpose was to review the scientific evidence on the effects of artificially sweetened beverage consumption on weight.

We included reviews that summarized data either quantitatively (with meta-analysis) or qualitatively (without meta-analysis).

Reviews of studies on beverages containing artificial sweeteners currently listed as Generally Recognized As Safe (GRAS) by the Food and Drug Administration [45] were included: acesulfame potassium (E950), aspartame (E951), salt of aspartame-acesulfame (E962), neotame (E961), saccharin (E954), sucralose (E955).

Reviews on studies of beverages containing artificial sweeteners previously approved, but currently not listed, as Generally Recognized As Safe (GRAS) by FDA were also included: sodium cyclamate, (E952, banned by FDA in 1969, currently in use in UK, EU and other countries), dulcin (banned by FDA in 1950), P-4000 (banned by FDA in 1950).

We only included reviews where full text was available in English; however, no non-English papers met the inclusion criteria.

A review was excluded if it assessed only the relationship between artificially sweetened beverage consumption and health effects other than weight outcomes such as cardiovascular disease, satiety, diabetes or cancer. We excluded reviews where the artificial sweeteners studied were only consumed in forms other than beverages. Only papers published in full were included; we excluded letters to the editor, editorials and published conference presentations.

### Search Methods for Identification Of Reviews

#### Electronic searches

We searched the following databases: Pubmed (1946–2014), Embase (1947–2014), Scopus (1823–2014), Web of Science (1840–2014), Sociological Abstracts (1952–2014). Data last updated: 22 August 2014.

#### Search strategy

We developed the search strategy with the assistance of a university librarian. We used the strategy shown in S1 Appendix for Pubmed and adapted it for the other databases.

#### Searching other resources

We also searched author files, reference lists of included papers and previous systematic reviews.

#### Selection of studies

**Fig 1:**
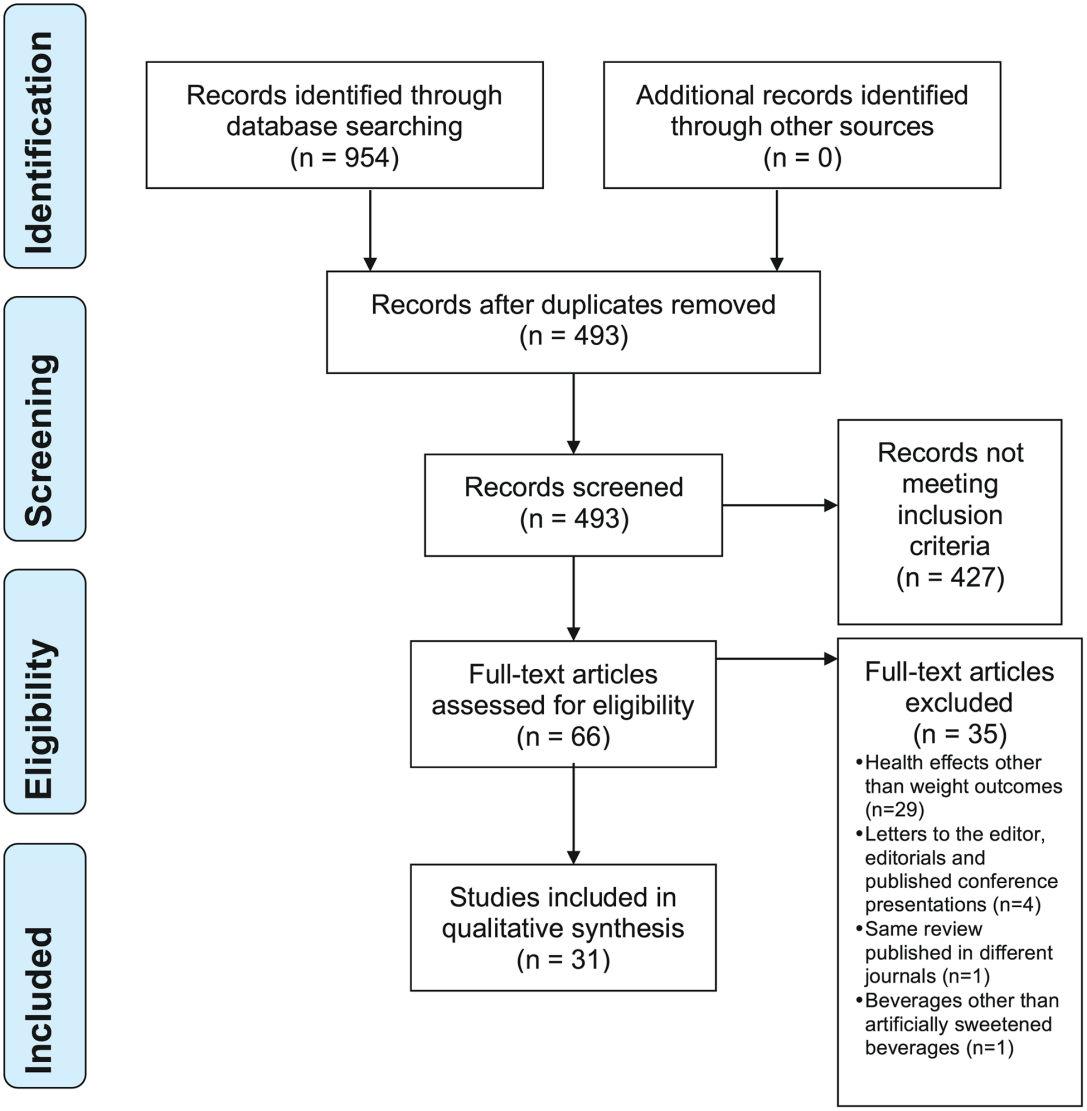
Prisma Flow Diagram

Two assessors (DM, CK) screened the titles and abstracts, when available, of all retrieved records for obvious exclusions, and assessed the full text of the remaining papers. Potentially eligible papers were sent to another assessor for final validation of the inclusion criteria. Any disagreement was resolved by consensus (DM, CK, LB). Details are reported in the “PRISMA Flow Chart” (Fig 1).

### Data Collection and Analysis

Two assessors (DM, CK) independently extracted data from included reviews on 1) review characteristics, 2) review outcomes, 3) risk of bias of the review 4) primary outcomes for this analysis: a) favorable results and b) favorable conclusions 5) review funding sources, 6) authors’ financial conflicts of interests, and 7) journal characteristics. Discrepancies were resolved by discussion with a third coder (LB). Unresolved discrepancies were coded as unclear. Data extracted and assessors evaluations for each review are reported in S2 Appendix.

### Review characteristics

- Artificial sweetener(s) studied
- Comparison group (s)
- Time period covered by studies included in the review
- Number of studies included in the review (if given, or number of articles if provided)
- Population studied (healthy adults; non healthy adults; children)
- Review included meta-analysis (yes/ no)

### Review outcomes

- Weight outcomes assessed (BMI; other measure of weight gain; other measure of obesity)
- Adverse outcomes assessed (diabetes; hypertension)

Weight and adverse outcomes were extracted only if specified by the authors in the text of the review.

### Risk of bias

To assess risk of bias in the included reviews, we used the criteria used for Cochrane reviews on funding bias[46]:

- whether explicit and “well defined” criteria that could be replicated by others were used to select studies for inclusion/exclusion in the review (yes/no/unclear)
- whether there was an adequate study inclusion method, with two or more assessors selecting studies (yes/no/unclear)
- whether the search for studies was comprehensive (yes/no/unclear)
- whether methodological differences and other characteristics that could introduce bias were controlled (yes/no/unclear)

Overall risk of bias was coded as 1) “low” if at least three of the four criteria were met. In any other case risk of bias was coded as 2) “high.”

### Primary Outcomes for this Analysis

- Results (favorable/unfavorable)
  First, results were extracted separately for each population studied (healthy adults; non healthy adults; children) in each review and coded as “positive” or “negative.”
  For each population results were defined as:
  - Positive: if more than 50 percent of the studies considered in the review reported that artificially sweetened beverage consumption in a population was associated with: a) statistically significant lower BMI score, overweight or obesity rate or no change compared to water; b) statistically significant lower BMI score, overweight or obesity rate compared to sugar-sweetened or other caloric beverages.
  - Negative: if more than 50 percent of the studies considered in the review reported that artificially sweetened beverage consumption in a population was associated with: a) statistically significant higher BMI score, overweight or obesity rate when compared to water; b) statistically significant higher BMI score, overweight or obesity rate or no change compared to sugar-sweetened or other caloric beverages.
  - Mixed: if the studies included in the review: a) did not report the results or 2) more than 50% of the studies did not fit the definition of “positive” or “negative”.
  Second, the overall review results were coded as “favorable” or “unfavorable”. Overall results were defined as:
  1. Favorable: if only positive results were reported for all the study populations considered in the review
  2. Unfavorable: if “negative” or “mixed” results were reported within one or more populations considered in the review. Mixed results were considered to be unfavorable based on the public health perspective which suggests that an exposure is unfavorable even if only one subpopulation is affected.
- Conclusions (favorable/unfavorable)
  Conclusions were defined as:
  1. Favorable: if authors stated or implied in their conclusions that a) artificially sweetened beverage consumption was definitely or probably associated with decreased weight, BMI, or obesity or no change, b) artificially sweetened beverages consumption was associated with increased weight, BMI, or obesity, but the association was attributed to poor study design or bias, or c) the evidence was inconclusive. Articles concluding that the evidence was inconclusive were classified as favorable because this conclusion is consistent with an acceptance of the null hypothesis that there is no relationship between artificially sweetened beverage consumption and increased BMI score, overweight or obesity rate.
  2. Unfavorable: if the authors stated or implied in their conclusions that artificially sweetened beverages were definitely or probably associated with increased weight, BMI or obesity.

### Review funding source

- Names of disclosed funding sources
- Type(s) of funding source disclosed were coded as 1) food industry (subcategories: artificial sweetener industry, cane and beet sugar industry, other food industry), 2) government, 3) private non-profit, 4) other, or 5) no funding disclosed.
  Organizations that could not be clearly classified into the above categories were coded based on the main funding sources for the organization, determined through a Google search.
- For reviews with a disclosed industry funder, the role of the funding source was coded as 1) not stated, 2) sponsor not involved, or 3) sponsor involved.

### Authors’ financial conflicts of interest

- Authors’ names
- Number of authors
- Number of authors with a financial conflict of interest (disclosed in the paper or not)
- Number of authors without a financial conflict of interest

Financial conflicts of interest of the review authors were coded as 1) yes, disclosed in the paper, 2) yes, not disclosed in the paper, 3) no disclosure statement, and 4) no financial conflicts of interest.

Financial conflicts of interest were defined per the July 2010 version of the International Committee of Medical Journal Editors [47] uniform disclosure form for potential conflicts of interest and included: current or former board membership, current or former consultancy work, current or former industry employment, expert testimony, industry grants (issued or pending), payment for lectures including service on speakers bureaus, payment for manuscript preparation, patents (planned, pending, or issued), royalties, payment for development of educational presentations, stock or stock options, and travel reimbursement, or other relations with relevant industries. For this study, relevant industries were defined as the food and beverage industry including trade groups and organizations such as the International Life Sciences Institute (ILSI).

We used a modification of a previous method to identify undisclosed financial conflicts of interest of authors [48]. For reviews where the authors did not provide a financial disclosure (“no disclosure statement”) or disclosed no financial conflict of interest (“no financial conflict of interest”), we searched the name of the authors in the European Food Safety Authority declaration of interests database, Center for Science in the Public Interest database, disclosures in other articles identified in Pubmed, Google, acknowledgments in other articles gathered for this study and their Curriculum Vitae. If a relevant conflict of interest was discovered using this search strategy, the search was discontinued and the author was classified as having a financial conflict of interest (“yes, not disclosed in the paper”).

### Journal characteristics

- Title
- Year and month published
- Journal name
- Peer-reviewed (yes/no)
- Journal funding (food industry/ mixed/ non-food industry)

Journal funding was defined as:

1. Food industry: if the journal or the scientific society associated with the journal was directly funded by the food and beverage industry, including trade groups and organizations such as the ILSI.
2. Mixed: if the journal or associated scientific society disclosed any funding or relationship (for example serving as press office or providing communication services other than advertising to companies) with the food industry.
3. Non-food industry: in any other case.

### Analysis

#### Primary analyses

1) We compared risk of bias and the number of favorable results and conclusions in reviews with artificial sweetener industry sponsorship to those with other sources of sponsorship. We hypothesized that reviews sponsored by the artificial sweetener industry would be more likely to have high risk of bias and results and conclusions that favored the artificial sweetener industry compared to reviews sponsored by other sources.

We conducted an *a priori* subgroup analysis comparing risk of bias, results and conclusions of reviews with different industry sponsors (for example, cane and beet sugar industry sponsored reviews vs artificial sweetener industry sponsored reviews).

2) We compared risk of bias, results and conclusions of reviews that had one or more authors with financial conflicts of interests to reviews that had authors with none. We hypothesized that reviews that had authors with financial conflicts of interest would be more likely to have high risk of bias and results and conclusions that favored the artificial sweetener industry compared to reviews that had authors with no financial conflicts of interest.

#### Secondary analyses

1) We also report the concordance between review results and conclusions as previous research has shown that industry sponsored studies are less likely to have results and conclusions that agree compared to non-industry sponsored studies [30]. We hypothesized that industry sponsorship and authors’ financial conflicts of interest would be associated with discordant results and conclusions (with results unfavorable toward artificially sweetened beverages and conclusions favorable toward artificially sweetened beverages).

2) We compared risk of bias, results and conclusions of reviews published in industry sponsored journals with those published in non-industry sponsored journals. We hypothesized that reviews published in industry sponsored journals would be more likely to have high risk of bias and results and conclusions that favored the artificial sweetener industry compared to reviews published in other journals.

#### Sensitivity analyses

Reviews with no disclosed funding sources may have been funded by industry. Therefore, we performed a sensitivity analysis excluding the reviews with no funding disclosed from the non-industry sponsored group.

#### Statistical analysis

Risk Ratios (RR) were calculated for all primary and secondary analyses with 95% confidence intervals (CI). Four reviews conducted meta-analyses, but we were unable to extract quantitative effect estimates that could be combined into a pooled effect size because of the different study designs and populations considered.

## Results

### Search results

After removal of duplicates, 493 references were identified and 31 reviews were included following full text screening (Fig 1, PRISMA Flow Diagram).

### Review characteristics

The 31 reviews were published between 1978 and 2014. Table 1 shows review characteristics by review funding source. Four reviews were funded by the artificial sweetener industry (ILSI, Ajinomoto Company, International Sweetener Association). Four were funded by the sugar or water industries and classified as competitor industries. Eleven disclosed non-industry funding and 13 disclosed no funding sources; these 2 groups were combined for analysis. Of the 18 reviews with disclosed funding sources, 10 stated that the sponsor was involved at some stage during the development of the manuscript.

**Table 1:** Review characteristics and risk of bias by funding source (n = 31).

REVIEW CHARACTERISTICS	FUNDING SOURCE						
	Total	As Producer	Sugar Industry	Other Industry	Government	Private Non- Profit	No Funding
	31	4	3	1	7	3	13
**General**							
Time period covered stated (years)	6	1	1	0	2	0	2
Number of studies included stated	6	2	1	1	2	0	0
Meta-analysis	4	2	0	1	1	0	0
**Weight Outcomes**							
BMI (kg/m2)	19	3	2	0	5	3	6
other measure of weight gain	31	4	3	1	7	3	13
other measure of obesity	19	2	2	0	5	3	7
**Adverse Outcomes**							
Diabetes	17	1	2	0	4	3	7
Hypertension	5	0	0	0	1	1	3
**Risk of Bias**							
Inclusion/Exclusion Criteria	9	2	1	1	2	2	1
Two or more assessors	2	0	0	0	2	0	0
Comprehensive Search Strategy	6	1	0	1	2	1	1
Methodological Discrepancies Explored	13	2	1	1	4	1	4
**Journal Characteristics**							
Peer-reviewed	27	3	3	1	7	3	10
Non peer-reviewed	4	1	0	0	0	0	3

Four reviews included meta-analyses. Only 6 reviews stated how many studies were included in the review. Weight outcomes assessed in the reviews include BMI, other measures of weight gain, and other measures of obesity. Adverse outcomes addressed in the reviews include diabetes (n = 17) and hypertension (n = 5).

Data extracted for each review are available in S2 Appendix.

#### Risk of bias

**Fig 2:**
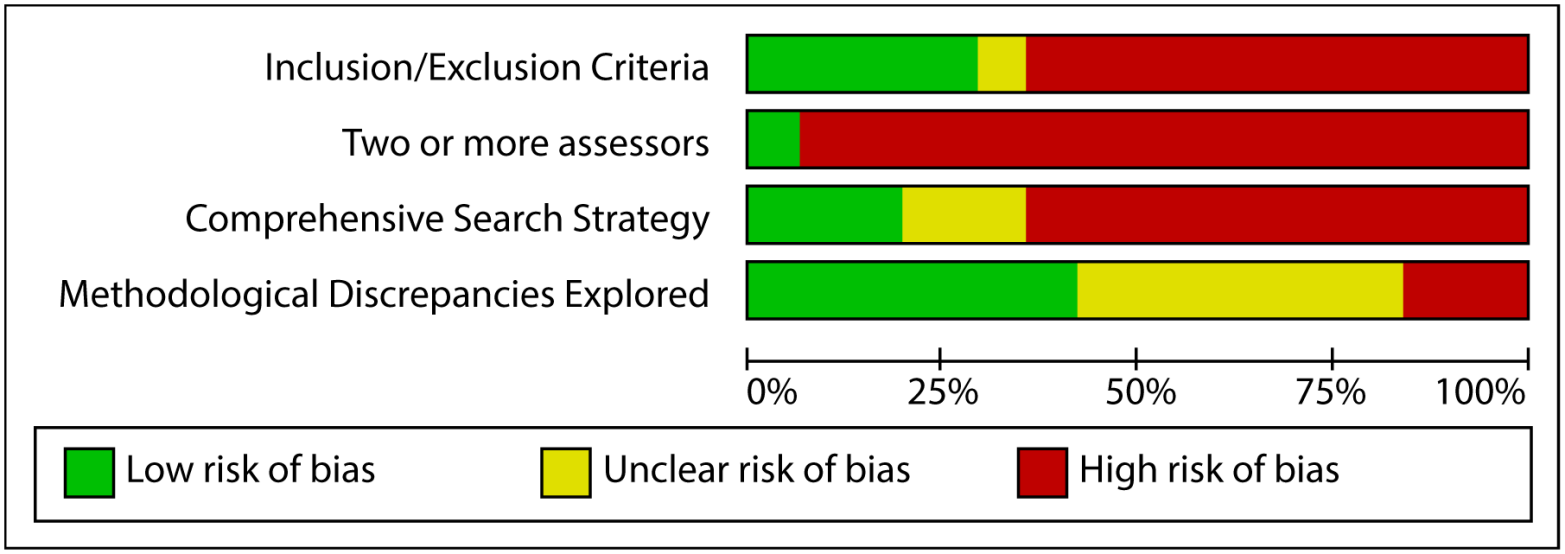
Risk of bias graph: review authors' judgments about each risk of bias item presented as percentages across all included studies

Agreement on coding of risk of bias by the two assessors was achieved for all reviews. Most of the included reviews had high or unclear risks of bias for each criterion (See Fig 2 and S3 Appendix). Eleven reviews reported their inclusion/exclusion criteria; only two reviews included two or more assessors for the evaluation of the evidence; six reviews included a comprehensive search strategy. The majority of the reviews (26/31) were coded as overall high risk of bias.

#### Journal characteristics

Twenty-seven reviews were published in peer-reviewed journals. We identified nineteen reviews published in non-industry funded journals and twelve reviews that were published in industry or mixed funded journals. Most reviews included studies on multiple artificial sweeteners; 6 included studies on only one artificial sweetener (5 on aspartame only, 1 saccharin only).

### Relationship between review sponsorship and risk of bias

The proportion of artificial sweetener industry sponsored reviews with overall high risk of bias (3/4) is similar to the proportion of non-industry sponsored reviews (included reviews with no funding disclosed) (20/23) RR: 0.86 (95% CI: 0.48 to 1.55) and competitor industry sponsored reviews (3/4) RR: 1.00 (95% CI: 0.44 to 2.22). We performed a sensitivity analysis excluding the reviews with no funding disclosed from the non-industry sponsored group. The proportion of artificial sweetener industry sponsored reviews with overall high risk of bias (3/4) remained similar to the proportion of government and private non-profit reviews with high risk of bias (6/10), RR: 1.25 (95% CI: 0.58 to 2.67).

### Relationship between review sponsorship and review results

The results, reported in Table 2 show that artificial sweetener industry sponsored reviews were more likely to have favorable results (3/4) than non-industry sponsored reviews (included reviews with no funding disclosed) (1/23), RR: 17.25 (95% CI: 2.34 to 127.29). We performed a sensitivity analysis excluding the reviews with no funding disclosed from the non-industry sponsored group. Artificial sweetener industry sponsored reviews still were more likely to have favorable results (3/4) than government and private no profit (1/10), RR: 7.5 (95% CI: 1.07 to 52.37).

**Table 2:** Results and conclusions of reviews by funding source (n = 31).

REVIEW OUTCOMES	FUNDING SOURCE						
	Total	As Producer	Sugar Industry	Other Industry	Government	Private Non- Profit	No Funding
	31	4	3	1	7	3	13
**Results**							
Favorable	4	3	0	0	1	0	0
Unfavorable	15	0	1	1	5	1	7
Unclear	12	1	2	0	1	2	6
**Conclusion**							
Favorable	19	4	0	0	4	3	8
Unfavorable	12	0	3	1	3	0	5

Then we compared results reported in artificial sweetener industry sponsored reviews with competitor industry sponsored reviews. A greater proportion of artificial sweetener industry sponsored reviews had favorable results (3/4) compared to competitor industry sponsored reviews (0/4).

We could not reach consensus on the results of 12 reviews. The results of these reviews were so confusing or were presented so incompletely that coding the results according to our criteria would require extensive interpretation by the authors (assessors evaluation reported in S2 Appendix). Therefore, these results were coded as “unclear”(see Methods). Industry sponsored (3/8) and non-industry sponsored (9/23) reviews had a similar risk of reporting unclear results RR: 0.70 (95% CI: 0.32 to 1.55).

### Relationship between review sponsorship and review conclusions

Artificial sweetener industry sponsored reviews were more likely to have favorable conclusions (4/4) than non-industry sponsored reviews (included reviews with no funding disclosed) (15/23), RR: 1.52 (95% CI: 1.14 to 2.06) (Table 2). We performed a sensitivity analysis excluding the reviews with no funding disclosed from the non-industry sponsored group. Artificial sweetener industry sponsored reviews were still more likely to have favorable conclusions (4/4) than government and private non-profit (7/10), although this was not statistically significant RR: 1.42 (95% CI: 0.95 to 2.14).

All four reviews funded by competitors of the artificial sweetener industry reported unfavorable conclusions (4/4) and all four reviews funded by the artificial sweetener industry had favorable conclusions, RR: ∞ (95% CI: NaN to ∞).

### Relationship between author financial conflict of interest and risk of bias

Reviews performed by authors with conflicts of interest had similar overall high risk of bias to reviews performed by authors without conflicts of interest RR: 0.92 (95% CI: 0.68 to 1.25). Eight of 9 reviews by authors without conflicts of interest had a high risk of bias, compared to 18 of 22 reviews by authors with conflicts of interest.

### Relationship between author financial conflicts of interest and review results

Table 3 summarizes the financial conflicts of interests of review authors. Authors of 42% (13/31) of reviews had conflicts of interest that were not disclosed in the article; most of these (n = 8) were in reviews that also had no disclosed funding sources. Most reviews were lacking disclosure statements for authors (19/31), regardless of whether the authors had a financial conflict of interest with the food industry (13/22) or not (6/9), with a similar likelihood of reporting between the two groups RR: 1.77 (95% CI: 0.66 to 4.76).

**Table 3:** Results and conclusions of reviews by conflicts of interest of authors (n = 31).

REVIEW OUTCOMES	AUTHORS CONFLICT OF INTEREST		
	Total	Industry	Non-Industry
	31	22	9
**Results**			
Favorable	4	4	0
Unfavorable	15	7	8
Unclear	12	11	1
**Conclusions**			
Favorable	19	18	1
Unfavorable	12	4	8

For 55% (17/31) of reviews, more than half of the authors had conflicts of interest with the food or beverage industry.

To test our hypothesis that reviews performed by authors with a conflict of interest with the food industry are more likely to report favorable results, we compared the 9 reviews including only authors that had no conflicts of interest with the food industry with 22 reviews performed by authors with disclosed or non-disclosed conflicts of interest (Table 4). None of the nine reviews performed by authors without conflicts of interest reported favorable results; whereas 4 reviews with authors with conflicts of interest had favorable results RR: ∞ (95% CI: NaN to ∞).

**Table 4:** Authors’ conflicts of interest by review funding source (n = 31).

REVIEW AUTHORS’ CONFLICTS OF INTEREST	FUNDING SOURCE						
	Total	As Producer	Sugar Industry	Other Industry	Government	Private Non- Profit	No Funding
	31	4	3	1	7	3	13
**Authors with COI**							
Industry (Disclosed)	9	4	2	1	1	1	0
Industry (Not Disclosed)	13	0	1	0	3	1	8
No disclosure	6	0	0	0	1	1	4
No conflict	3	0	0	0	2	0	1
**Proportion of Authors with COI**							
< = 50%	5	0	0	1	1	2	1
>50%	17	4	3	0	3	0	7
No disclosure or conflict	9	0	0	0	3	1	5

Reviews performed by authors with a conflict of interest with the food industry were more likely to report unclear results (11/22) than reviews performed by authors without conflicts of interest (1/8) RR: 4.50 (95% CI: 0.68 to 29.92).

### Relationship between author financial conflict of interest and review conclusions

Reviews performed by authors with a conflict of interest with the food industry were more likely to have favorable conclusions (18/22) than reviews performed by authors without conflicts of interest (4/9), RR: 7.36 (95% CI: 1.15 to 47.22). Notably, the only reviews performed by authors with conflicts of interest that reported unfavorable conclusions were all funded by competitor industries (4/4).

### Relationship of author financial conflicts of interest with concordance of review results and conclusion

All reviews that reported favorable conclusions and discordant (unfavorable) results were conducted by authors with conflicts of interest (5/5). None of these five reviews were sponsored by industry. All the reviews that reported favorable conclusions and concordant (favorable) results were performed by authors with financial conflicts of interest (4/4) and the majority were funded by the artificial sweetener industry (3/4). Of the ten reviews that reported unfavorable conclusions and concordant (unfavorable) results, eight were prepared by authors without conflicts of interest and two by authors with conflicts of interest (and funded by artificial sweetener competitor industries). Reviews by authors with conflicts of interest were less likely to have concordant results and conclusion than reviews by authors without conflicts of interest RR: 0.55 (95% CI: 0.32 to 0.94) (Table 3).

The relationship of source of sponsorship and concordance of review results and conclusion could not be calculated because of the small samples size and the high number of reviews with unclear results sponsored by the artificial sweetener (1/4) and competitor (2/4) industries (Table 2).

### Relationship between journal funding and risk of bias

Reviews published in industry or mixed funding journals had similar overall high risk of bias to reviews published in non-industry funded journals RR: 0.92 (95% CI: 0.68 to 1.25).

### Relationship between Journal Funding and Review Results

Only one review published in a non-industry funded journal reported favorable results (1/19) compared to 3/12 reviews published in journals with industry or mixed funding (Table 5). Reviews published in journals with industry or mixed funding were as likely to report favorable results (3/12) as reviews published in non-industry funded journals (1/19) RR: 4.75 (95% CI: 0.56 to 40.56).

**Table 5:** Results and conclusions of reviews by journal funding (n = 31).

REVIEW OUTCOMES	JOURNAL FUNDING			
	Total	Industry	Mixed	Non-Industry
	31	4	8	19
**Results**				
Favorable	4	0	3	1
Unfavorable	15	2	1	12
Unclear	12	2	4	6
**Conclusions**				
Favorable	19	3	8	8
Unfavorable	12	1	0	11

### Relationship between journal funding and review conclusions

Almost all the reviews published in industry or mixed funded journals reported favorable conclusions (11/12) (Table 5). Reviews published in industry and mixed funded journals more often had favorable conclusions (11/12) than reviews published in non-industry funded journals (8/19) RR: 2.18 (95% CI: 1.25 to 3.79).

## Discussion

Our findings show that review sponsorship, financial conflicts of interests of authors and journal funding are all associated with favorable outcomes related to the effects of artificially sweetened beverages on weight outcomes. Reviews sponsored by the artificial sweetener industry were more likely to report results and conclusions that favored artificially sweetened beverages than non-industry sponsored reviews. We also found that reviews performed by authors with a conflict of interest with the food industry were more likely to have results and conclusions that favored artificially sweetened beverages than reviews performed by authors without financial conflicts of interest. Reviews performed by authors with financial conflicts of interest also were more likely to report unclear results and to lack concordance between results and conclusions. The lack of concordance was primarily due to the reviews having favorable conclusions when the results were unclear or not favorable. Thus, authors with financial conflicts of interest with the food industry were more likely to put a positive “spin” on the conclusions of their reviews [49].

Our study is consistent with similar recent findings that the main global sweetener producer, the cane and beet sugar industry, also supports research that favors their product. Reviews of the health effects of sugar sweetened beverages performed by authors with financial ties to the sugar industry are five times more likely to conclude there is no association of sugar consumption with weight gain [42]. Our results are also in line with the consolidated evidence that industry sponsorship of various types of research is associated with favorable outcomes for the sponsor in both human [29, 31–34] and animal studies[35, 36].

We also found that reviews performed by artificial sweetener industry competitors (e.g. the sugar industry) were more likely to have unfavorable results and conclusions on the effects of artificially sweetened beverages on weight than artificial sweetener industry sponsored reviews and non-industry sponsored reviews. These findings suggest that fair evaluations of safety and effectiveness of products might be potentially undermined by reviews on a product performed by competitors. Therefore, competitor funding should be considered as a source of bias in reviews of a product.

Moreover, we showed that reviews published in journals funded partially or in full by the food industry more often have conclusions that are favorable towards artificially sweetened beverages than reviews published in non-industry funded journals. The role of the funding of journals and its effects on study outcomes has been rarely investigated, and early (and recent) warnings on the risk of transforming healthcare, environmental and toxicological journals into marketing instruments have been largely ignored [50–53].

Notably, the differences in the results and conclusions that we observed related to sponsorship, author conflicts of interest and journal funding cannot be explained by differences in the risks of bias in how the reviews were conducted. Risk of bias of the reviews was high in all comparison groups. To reduce bias, reviews of the effects of artificial sweeteners on weight need improvement in their search strategies, data collection and assessment of included studies. In addition, the majority of the reviews were published in peer-reviewed journals. Although peer review can be a valuable tool for increasing the quality of scientific publication [54], it is not sufficient to mitigate the observed influence of study sponsorship, conflict of interest and journal funding on review results and conclusions.

Almost half of the reviews had authors that failed to disclose relevant conflicts of interest with the food industry. This lack of disclosure is consistent with the lack of transparency and compliance with conflict of interest disclosure policies reported in several fields [48, 55–58]. Thus, disclosures in journals do not give an accurate assessment of authors’ conflicts of interest. Further investigation of authors’ financial ties using different databases, disclosures made in other research articles, and additional resources may be necessary to provide a more accurate description than a single declaration of interest within a single publication. In addition, journals need to enforce their disclosure requirements and penalize authors who fail to disclose. The WHO/IARC, the US Institute of Medicine, the ICMJE and the Collegium Ramazzini have all pointed out the need for transparency and accountability in policies for declaration of interest [47, 59–62]. The US National Library of Medicine was also recently urged to include information about authors’ competing interests in the abstracts of articles submitted to PubMed, the library’s online database of biomedical literature [63].

Our systematic review had limitations related to the conduct and reporting of the included reviews, as well as the small sample size of studies available. Our analysis relies on how included reviews defined “funding” and “financial conflict of interest of the authors” and we cannot exclude the possibility that other financial interests and other sources of funding were present. In addition, we categorized industry funding into artificial sweetener industry, competitor industry and other industry. This is necessary because the interests of these different corporate sponsors differ, but it resulted in small number of reviews in each category. Also, categorizing funding sources in the food industry is complicated. For example, we categorized the American Diabetes Association as non-profit. However, the ADA is supported by a variety of companies (e.g. Johnson and Johnson, Nutrisystem) that produce or sell artificial sweeteners, as reported also by CSPI [64–66]. We conducted additional analyses where we categorized the American Diabetes Association as the artificial sweetener industry and this did not change the observed association of artificial sweetener industry funding with the results or conclusions of the reviews.

There is increasing momentum towards the inclusion of funding source and author conflicts of interest as a risk of bias domain for evidence-based evaluation. The Navigation Guide already includes both as a risk of bias domain in both human and animal studies [67, 68], while Cochrane and GRADE are considering it, but have not yet adopted it [69]. According to the US Agency for Healthcare Research and Quality and the US National Toxicology Program, funding source is recommended as a factor to consider when evaluating risk of bias of individual studies for selective reporting and then again for evaluating the body of evidence for publication bias [67, 68, 70]. The US Food and Drug Administration and the European Food Safety Authority, that evaluate safety and risks of food consumption, both pre and post marketing, including effects on weight of sweeteners, and eventually establish an Acceptable Daily Intake (ADI) [71–73] were recently criticized for the number of panelists with financial interests raising concerns on the integrity and transparency of their evaluation processes [74–76]. The Advisory Committee of the Dietary Guidelines for Americans 2015–2020 considered several reviews, but did not assess funding source as a risk of bias [7]. Our results confirm that funding source and conflicts of interest are a source of bias in reviews that cannot be detected by other risk of bias rating criteria.

## Conclusion

Our systematic review shows that financial conflicts of interest introduced a bias at all levels of the research and publication process (author financial ties, review sponsorship and journal funding), affecting the outcomes of reviews and possibly undermining the quality and transparency of public health evaluations that are reliant on these reviews. The bias introduced by financial interests could not be ascribed to the overall risk of bias of the reviews and was not prevented by the peer review process.

## Supporting Information

S1 Appendix Search Strategy. (DOCX)

S2 Appendix Database. (XLS)

S3 Appendix Risk of Bias. (DOCX)

S1 File List of Excluded Studies. (DOCX)

S2 File PRISMA Checklist. (DOC)
