# Correction: Relationship between Research Outcomes and Risk of Bias, Study Sponsorship, and Author Financial Conflicts of Interest in Reviews of the Effects of Artificially Sweetened Beverages on Weight Outcomes: A Systematic Review of Reviews

**DOI:** 10.1371/journal.pone.0230469

**Published:** 2020-03-10

**Authors:** Daniele Mandrioli, Cristin E Kearns, Lisa A. Bero

There is a typographical error in the listed ratio of favorable conclusions without conflicts of interest. The text is changed from 4/9 to the correct ratio of 1/9. The following sentences need to be amended, changed number bolded below:

- The final sentence of the “Results” subsection of the Abstract: Reviews performed by authors that had a financial conflict of interest with the food industry (disclosed in the article or not) were more likely to have favorable conclusions (18/22) than reviews performed by authors without conflicts of interest (**1/9**), RR: 7.36 (95% CI: 1.15 to 47.22). Risk of bias was similar and high in most of the reviews.
- The “Relationship between author financial conflict of interest and review conclusions” subsection of the Results: Reviews performed by authors with a conflict of interest with the food industry were more likely to have favorable conclusions (18/22) than reviews performed by authors without conflicts of interest (**1/9**), RR: 7.36 (95% CI: 1.15 to 47.22). Notably, the only reviews performed by authors with conflicts of interest that reported unfavorable conclusions were all funded by competitor industries (4/4).

The authors note that the correct number is shown in Table 3 and was used in their calculations of relative risk.
