# Supplementary material for: Relationship between Research Outcomes and Risk of Bias, Study Sponsorship, and Author Financial Conflicts of Interest in Reviews of the Effects of Artificially Sweetened Beverages on Weight Outcomes: A Systematic Review of Reviews
Source: PLoS One. 2016 Sep 8;11(9):e0162198. doi: 10.1371/journal.pone.0162198 (PMC5015869; doi:10.1371/journal.pone.0162198)
Supplement: S1 Appendix — (DOCX) [file pone.0162198.s001.docx]

**Mandrioli, Kearns, Bero Completed date: August 22, 2014**

**Literature search**

Investigate whether industry sponsored studies of artificially sweetened beverage consumption and its effects on weight have different outcomes and risk of bias, compared with studies having other sources of sponsorship

**Summary**

**Databases Citation counts Coverage dates**

PubMed (NLM) 176 1946 +

Embase (Elsevier) 326 1947 +

Scopus 357 1823 +

Web of Science (Thomson Reuters) 95 *

Sociological Abstracts 0 1952 +

***** Web of Science Core Collection: Citation Indexes

- Science Citation Index Expanded (SCI-EXPANDED) --1965-present
- Social Sciences Citation Index (SSCI) --1900-present
- Arts & Humanities Citation Index (A&HCI) --1975-present
- Conference Proceedings Citation Index- Science (CPCI-S) --1990-present
- Conference Proceedings Citation Index- Social Science & Humanities (CPCI-SSH) --1990-present
- Book Citation Index– Science (BKCI-S) --2005-present
- Book Citation Index– Social Sciences & Humanities (BKCI-SSH) --2005-present

#### Web of Science Core Collection: Chemical Indexes

- Current Chemical Reactions (CCR-EXPANDED) --1985-present
  *(Includes Institut National de la Propriete Industrielle structure data back to 1840)*
- Index Chemicus (IC) --1993-present

Data last updated: 2014-08-21

**PubMed(NLM), run date 8/22/14**

(sweetening agents[mh:noexp] OR “sweetening agent” OR “sweetening agents”[tiab] OR non-nutritive sweetener* OR nonnutritive sweetener* OR acesulfame OR “acesulfame k” OR acetosulfame OR aspartame OR cyclamate OR dulcin OR neotame OR saccharin OR sucralose OR artificial sweetener* OR "artificially sweetened" OR high intensity sweetener* OR high potency sweetener* OR intense sweetener* OR low calorie sweetener* OR “no calorie” OR “reduced sugar” OR "sugar free" OR sugar substitute* OR diet beverage* OR diet drink* OR diet soda* OR diet soft drink* OR ((“non-caloric” OR noncaloric OR “non-calorie” OR “non-nutritive” OR nonnutritive OR reduced calorie* ) AND (beverage* OR drink* OR soda* OR soft drink*))) AND ("abdominal fat" OR "adipose tissue" OR adiposity OR "body fat" OR "body mass" OR "body mass index" OR BMI[tiab] OR "fat mass" OR "intraabdominal fat" OR obese OR obesity OR overweight OR "visceral fat" OR "waist circumference" OR weight) AND (review OR reviews) NOT ((animals[mh] NOT humans[mh]) OR mice[ti] OR mouse[ti] OR rat[ti] OR rats[ti])

176 refs

Search notes: "on line" = "on-line"

**Embase (Elsevier), run date 8/22/14**

('sweetening agent'/de or sweetening next/1 agent* or 'non-nutritive sweetener' or 'non-nutritive sweeteners' OR 'nonnutritive sweetener' or 'nonnutritive sweeteners' or acesulfame or 'acesulfame k' or acetosulfame or aspartame or cyclamate or dulcin or neotame or saccharin or sucralose or 'artificial sweetener' or 'artificial sweeteners' or 'artificially sweetened' or 'high intensity sweetener' or 'high intensity sweeteners' or 'high potency sweetener' or 'high potency sweeteners' or 'intense sweetener' or 'intense sweeteners' or 'low calorie sweetener' OR 'low calorie sweeteners' OR ‘no calorie” OR “reduced sugar” OR ‘sugar free” OR ‘sugar substitute’ OR ‘sugar substitutes’ or 'diet beverage' OR 'diet beverages' OR 'diet drink' OR 'diet drinks' OR 'diet soda' OR 'diet sodas' OR 'diet soft drink' OR 'diet soft drinks' OR (('non-caloric' OR noncaloric OR 'non-calorie' OR 'non-nutritive' OR nonnutritive OR 'reduced calorie' OR ‘reduced calories’) AND (beverage* OR drink OR drinks OR soda OR sodas OR ‘soft drink’ OR ‘soft drinks’)) ) AND ('abdominal fat' or 'adipose tissue' or adiposity or 'body fat' or 'body mass'/de or 'body mass':ab,ti or 'body mass index' or bmi:ab,ti or 'fat mass' or 'intraabdominal fat' or obese or obesity or overweight or 'visceral fat' or 'waist circumference' or weight) and (review or reviews) not ([animals]/lim not ([humans]/lim or 'patients'/exp) or mice:ti or mouse:it or rat:ti or rats:ti)

326 refs

Search notes: "on line" = "on-line"

**Scopus, run date 8/22/14**

((TITLE-ABS-KEY("sweetening agent" OR "sweetening agents" OR "non-nutritive sweetener" OR "non-nutritive sweeteners" OR "nonnutritive sweetener" OR "nonnutritive sweeteners" OR acesulfame OR "acesulfame k" OR acetosulfame OR aspartame OR cyclamate OR dulcin OR neotame OR saccharin OR sucralose OR "artificial sweetener" OR "artificial sweeteners" OR "artificially sweetened" OR "high intensity sweetener" OR "high intensity sweeteners" OR "high potency sweetener" OR "high potency sweeteners" OR "intense sweetener" OR "intense sweeteners" OR "low calorie sweetener" OR "low calorie sweeteners" OR "no calorie" OR "reduced sugar" OR "sugar free" OR "sugar substitute" OR "sugar substitutes" OR "diet beverage" OR "diet beverages" OR "diet drink" OR "diet drinks" OR "diet soda" OR "diet sodas" OR "diet soft drink" OR "diet soft drinks" OR (('non-caloric' OR noncaloric OR 'non-calorie' OR 'non-nutritive' OR nonnutritive OR 'reduced calorie' OR 'reduced calories') AND (beverage* OR drink OR drinks OR soda OR sodas OR "soft drink" OR "soft drinks")))) AND (TITLE-ABS-KEY("abdominal fat" OR "adipose tissue" OR adiposity OR "body fat" OR "body mass" OR "body mass index" OR bmi OR "fat mass" OR "intraabdominal fat" OR obese OR obesity OR overweight OR "visceral fat" OR "waist circumference" OR weight)) AND (TITLE-ABS-KEY(review OR reviews))) AND NOT (TITLE(animal* OR mice OR mouse OR rat OR rats))

357 refs

Search notes:

"on line" = "on-line"

No human/animal filter

Subject coverage:

[Life Sciences](javascript:void(0)) (> 4,300 titles.)

[Health Sciences](javascript:void(0)) (> 6,800 titles. 100% Medline coverage)

[Physical Sciences](javascript:void(0)) (> 7,200 titles.)

[Social Sciences & Humanities](javascript:void(0)) (> 5,300 titles.)

Bottom of Form

Top of Form

**Web of Science (Thomson Reuters), 8/22/14**

| **Set** | **Results** |  |
| --- | --- | --- |
| # 6 | [95](http://apps.webofknowledge.com/summary.do?product=WOS&doc=1&qid=37&SID=4Djm4k2cMnklMUsXr8H&search_mode=AdvancedSearch) | #4 NOT #5  Indexes=SCI-EXPANDED, SSCI, A&HCI, CPCI-S, CPCI-SSH, BKCI-S, BKCI-SSH, CCR-EXPANDED, IC Timespan=All years |
| # 5 | [1,528,069](http://apps.webofknowledge.com/summary.do?product=WOS&doc=1&qid=25&SID=4Djm4k2cMnklMUsXr8H&search_mode=GeneralSearch) | **TITLE:** (animal* OR mice OR mouse OR rat OR rats) |
| # 4 | [98](http://apps.webofknowledge.com/summary.do?product=WOS&doc=1&qid=36&SID=4Djm4k2cMnklMUsXr8H&search_mode=CombineSearches) | #3 AND #2 AND #1 |
| # 3 | [1,463,805](http://apps.webofknowledge.com/summary.do?product=WOS&doc=1&qid=23&SID=4Djm4k2cMnklMUsXr8H&search_mode=GeneralSearch) | **TOPIC:** (review or reviews) |
| # 2 | [1,383,904](http://apps.webofknowledge.com/summary.do?product=WOS&doc=1&qid=22&SID=4Djm4k2cMnklMUsXr8H&search_mode=AdvancedSearch) | TS=("abdominal fat" OR "adipose tissue" OR adiposity OR "body fat" OR "body mass" OR "body mass index" OR bmi OR "fat mass" OR "intraabdominal fat" OR obese OR obesity OR overweight OR "visceral fat" OR "waist circumference" OR weight) |
| # 1 | [9,461](http://apps.webofknowledge.com/summary.do?product=WOS&doc=1&qid=35&SID=4Djm4k2cMnklMUsXr8H&search_mode=AdvancedSearch) | TS=("sweetening agent" OR "sweetening agents" OR "non-nutritive sweetener" OR "non-nutritive sweeteners" OR "nonnutritive sweetener" OR "nonnutritive sweeteners" OR acesulfame OR “acesulfame k” OR acetosulfame OR aspartame OR cyclamate OR dulcin OR neotame OR saccharin OR sucralose OR "artificial sweetener" OR "artificial sweeteners" OR "artificially sweetened" OR "high intensity sweetener" OR "high intensity sweeteners" OR "high potency sweetener" OR "high potency sweeteners" OR "intense sweetener" OR "intense sweeteners" OR "low calorie sweetener" OR "low calorie sweeteners" OR "no calorie" OR "reduced sugar" OR "sugar free" OR "sugar substitute" OR "sugar substitutes" OR “diet beverage” OR “diet beverages” OR “diet drink” OR “diet drinks” OR “diet soda” OR “diet sodas” OR “diet soft drink” OR “diet soft drinks” OR (('non-caloric' OR noncaloric OR 'non-calorie' OR 'non-nutritive' OR nonnutritive OR 'reduced calorie' OR ‘reduced calories’) AND (beverage* OR drink OR drinks OR soda OR sodas OR “soft drink” OR “soft drinks”)) )  Indexes=SCI-EXPANDED, SSCI, A&HCI, CPCI-S, CPCI-SSH, BKCI-S, BKCI-SSH, CCR-EXPANDED, IC Timespan=All years |

Search notes:

“on line” = “on-line”

Use double quotes around phrases!

No human/animal filter

**Social Sciences Abstracts (ProQuest), 8/22/14**

("sweetening agent" OR "sweetening agents" OR "non-nutritive sweetener" OR "non-nutritive sweeteners" OR "nonnutritive sweetener" OR "nonnutritive sweeteners" OR acesulfame OR "acesulfame k" OR acetosulfame OR aspartame OR cyclamate OR dulcin OR neotame OR saccharin OR sucralose OR "artificial sweetener" OR "artificial sweeteners" OR "artificially sweetened" OR "high intensity sweetener" OR "high intensity sweeteners" OR "high potency sweetener" OR "high potency sweeteners" OR "intense sweetener" OR "intense sweeteners" OR "low calorie sweetener" OR "low calorie sweeteners" OR "no calorie" OR "reduced sugar" OR "sugar free" OR "sugar substitute" OR "sugar substitutes" OR "diet beverage" OR "diet beverages" OR "diet drink" OR "diet drinks" OR "diet soda" OR "diet sodas" OR "diet soft drink" OR "diet soft drinks" OR (('non-caloric' OR noncaloric OR 'non-calorie' OR 'non-nutritive' OR nonnutritive OR 'reduced calorie' OR 'reduced calories') AND (beverage* OR drink OR drinks OR soda OR sodas OR "soft drink" OR "soft drinks")) ) AND ("abdominal fat" OR "adipose tissue" OR adiposity OR "body fat" OR "body mass" OR "body mass index" OR bmi OR "fat mass" OR "intraabdominal fat" OR obese OR obesity OR overweight OR "visceral fat" OR "waist circumference" OR weight) AND (review OR reviews) NOT TI (animal* OR mice OR mouse OR rat OR rats)

0 ref
