# Supplementary material for: Relationship between Research Outcomes and Risk of Bias, Study Sponsorship, and Author Financial Conflicts of Interest in Reviews of the Effects of Artificially Sweetened Beverages on Weight Outcomes: A Systematic Review of Reviews
Source: PLoS One. 2016 Sep 8;11(9):e0162198. doi: 10.1371/journal.pone.0162198 (PMC5015869; doi:10.1371/journal.pone.0162198)

Figure 2. Risk of bias summary: review authors' judgements about each risk of bias item for each included study.


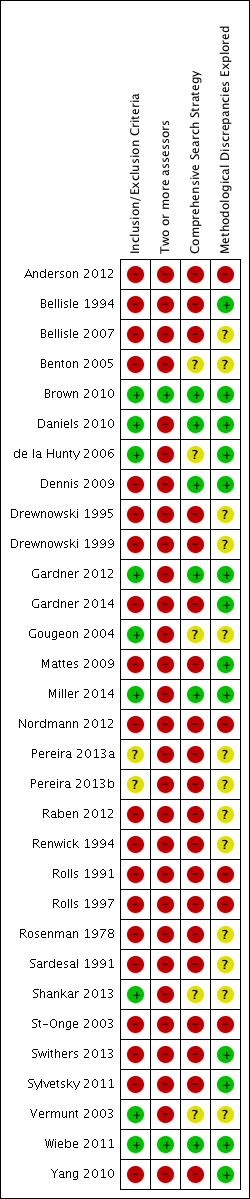

Supplement: S3 Appendix — (DOCX) [file pone.0162198.s003.docx]
