# Supplementary material for: Relationship between Research Outcomes and Risk of Bias, Study Sponsorship, and Author Financial Conflicts of Interest in Reviews of the Effects of Artificially Sweetened Beverages on Weight Outcomes: A Systematic Review of Reviews
Source: PLoS One. 2016 Sep 8;11(9):e0162198. doi: 10.1371/journal.pone.0162198 (PMC5015869; doi:10.1371/journal.pone.0162198)
Supplement: S1 File — (DOCX) [file pone.0162198.s004.docx]

**RECORDS NOT MEETING INCLUSION CRITERIA (n=427)**

1. The cyclamate story unfolds. Food Cosmet Toxicol. 1970;8(5):563-5. Epub 1970/10/01. PubMed PMID: 4923933.

2. Dietary recommendations for diabetics for the 1980s--a policy statement by the British Diabetic Association: prepared by the Nutrition Sub-Committee of the British Diabetic Association's Medical Advisory Committee. Human nutrition Applied nutrition. 1982;36(5):378, 80, 82 passim. Epub 1982/10/01. PubMed PMID: 6292137.

3. Nutrition recommendations and principles for people with diabetes mellitus. Journal of the American Dietetic Association. 1994;94(5):504-6.

4. Nutrition recommendations and principles for people with diabetes mellitus. Diabetes Care. 1999;22(1 SUPPL.):S42-S5.

5. Nutrition recommendations and principles for people with diabetes mellitus. Diabetes Care. 2000;23(1 SUPPL.):S43-S6.

6. NTP-CERHR Expert Panel report on the reproductive and developmental toxicity of methanol. Reproductive Toxicology. 2004;18(3):303-90.

7. Nutrition recommendations and interventions for diabetes: A position statement of the American Diabetes Association. Diabetes Care. 2007;30(SUPPL. 1):S48-S65.

8. International Journal of Obesity: Introduction. International Journal of Obesity. 2009;33(SUPPL. 3):S1-S88.

9. Ahmed FE. Effect of diet, life style, and other environmental/chemopreventive factors on colorectal cancer development, and assessment of the risks. Journal of Environmental Science and Health Part C-Environmental Carcinogenesis & Ecotoxicology Reviews. 2004;22(2):91-147. doi: 10.1081/lesc-200038263. PubMed PMID: WOS:000225521000002.

10. Ahmed FE, Thomas DB. Assessment of the carcinogenicity of the nonnutritive sweetener cyclamate. Crit Rev Toxicol. 1992;22(2):81-118. Epub 1992/01/01. doi: 10.3109/10408449209146307. PubMed PMID: 1510820.

11. Akram M, Hamid A. Mini review on fructose metabolism. Obesity Research and Clinical Practice. 2013;7(2):e89-e94.

12. Al-Waili N, Salom K, Al-Ghamdi A, Ansari MJ, Al-Waili A, Al-Waili T. Honey and Cardiovascular Risk Factors, in Normal Individuals and in Patients with Diabetes Mellitus or Dyslipidemia. Journal of Medicinal Food. 2013;16(12):1063-78. doi: 10.1089/jmf.2012.0285. PubMed PMID: WOS:000328457600001.

13. Allison DB, Mattes RD. Nutritively sweetened beverage consumption and obesity: The need for solid evidence on a fluid issue. JAMA - Journal of the American Medical Association. 2009;301(3):318-20. doi: 10.1017/S1368980008002644Vartanian, L.R., Schwartz, M.B., Brownell, K.D., Effects of soft drink consumption on nutrition and health: A systematic review and meta-analysis (2007) Am J Public Health, 97 (4), pp. 667-675; Nagourney, E., Nutrition: Study links sugary drinks to teenagers' weight New York Times, , http://www.nytimes.com/2006/03/07/health/07nutr.html?fta=y, March 7, 2006, Accessed October 31, 2008; Soft drinks weight gain warning, , http://news.bbc.co.uk/1/hi/health/4778436.stm, March 6, 2006, Accessed December 7, 2008.

14. Ambrosini GL, Oddy WH, Huang RC, Mori TA, Beilin LJ, Jebb SA. Spective associations between sugar-sweetened beverage intakes and cardiometabolic risk factors in adolescents. American Journal of Clinical Nutrition. 2013;98(2):327-34.

15. Amon I, Amon K. Passage of drugs into the amniotic fluid. Zentralblatt fur Gynakologie. 1976;98(16):961-9.

16. Anderson GH. Sugars-containing beverages and post-prandial satiety and food intake. International Journal of Obesity. 2006;30:S52-S9. doi: 10.1038/sj.ijo.0803493. PubMed PMID: WOS:000242337600010.

17. Anderson GH, Woodend D. Consumption of sugars and the regulation of short-term satiety and food intake. American Journal of Clinical Nutrition. 2003;78(4):843S-9S. PubMed PMID: WOS:000185403700029.

18. Andreatta MM, Navarro A, Eynard AR. Urinary tract tumors, biology and risk for artificial sweeteners use with particular emphasis on some South American countries. Current Nutrition and Food Science. 2008;4(3):185-95.

19. Arcella D, Le Donne C, Piccinelli R, Leclercq C. Dietary estimated intake of intense sweeteners by Italian teenagers. Present levels and projections derived from the INRAN-RM-2001 food survey. Food and Chemical Toxicology. 2004;42(4):677-85.

20. Argiri K, Sotiropoulos A, Psarou E, Taouxis M, Zampelas A, Kapsokefalou M. A series of deserts formulated for diabetic patients affect favorably their postprandial glucose and insulin response. Obesity Reviews. 2011;12:228.

21. Armstrong LE. Challenges of linking chronic dehydration and fluid consumption to health outcomes. Nutrition Reviews. 2012;70:S121-S7. doi: 10.1111/j.1753-4887.2012.00539.x. PubMed PMID: WOS:000310601700005.

22. Armstrong LE, Barquera S, Duhamel JF, Hardinsyah R, Haslam D, Lafontan M. Recommendations for healthier hydration: Addressing the public health issues of obesity and type 2 diabetes. Clinical Obesity. 2012;2(5-6):115-24.

23. Asenso Barnieh B. Satisfying sugar cravings in people who are obese and diabetic with synsepalum dulcificum: Underutilized and neglected fruit specie in Ghana. Annals of Nutrition and Metabolism. 2013;63:1144.

24. Astrup A. Carbohydrates as macronutrients in relation to protein and fat for body weight control. International Journal of Obesity. 2006;30(SUPPL. 3):S4-S9.

25. Astrup A, Dyerberg J, Selleck M, Stender S. Nutrition transition and its relationship to the development of obesity and related chronic diseases. Obesity Reviews. 2008;9(SUPPL. 1):48-52.

26. Aydin S, Aksoy A, Aydin S, Kalayci M, Yilmaz M, Kuloglu T, et al. Today's and yesterday's of pathophysiology: biochemistry of metabolic syndrome and animal models. Nutrition. 2014;30(1):1-9. Epub 2013/12/03. doi: 10.1016/j.nut.2013.05.013. PubMed PMID: 24290591.

27. Bachman CM, Baranowski T, Nicklas TA. Is there an association between sweetened beverages and adiposity? Nutrition Reviews. 2006;64(4):153-74.

28. Bairagi A, Sarkar Ghosh K, Sen SK, Ray AK. Duckweed (Lemna polyrhiza) leaf meal as a source of feedstuff in formulated diets for rohu (Labeo rohita Ham.) fingerlings after fermentation with a fish intestinal bacterium. Bioresource Technology. 2002;85(1):17-24.

29. Bantle JP. Is fructose the optimal low glycemic index sweetener? Nestlé Nutrition workshop series Clinical & performance programme. 2006;11:83-91; discussion 2-5.

30. Barnard ND. Trends in food availability, 1909-2007 1-3. American Journal of Clinical Nutrition. 2010;91(5):1530S-6S.

31. Barquera S, Campos I, Rivera JA. Mexico attempts to tackle obesity: the process, results, push backs and future challenges. Obesity reviews : an official journal of the International Association for the Study of Obesity. 2013;14 Suppl 2:69-78. Epub 2013/10/10. doi: 10.1111/obr.12096. PubMed PMID: 24103026.

32. Basaranoglu M, Basaranoglu G, Sabuncu T, Senturk H. Fructose as a key player in the development of fatty liver disease. World journal of gastroenterology : WJG. 2013;19(8):1166-72. Epub 2013/03/14. doi: 10.3748/wjg.v19.i8.1166. PubMed PMID: 23482247; PubMed Central PMCID: PMCPmc3587472.

33. Basciano H, Federico L, Adeli K. Fructose, insulin resistance, and metabolic dyslipidemia. Nutrition and Metabolism. 2005;2.

34. Beereboom JJ. Low calorie bulking agents. CRC critical reviews in food science and nutrition. 1979;11(4):401-13.

35. Behrendt W, Surmann M. Posttraumatic hypocaloric parenteral nutrition - Development and clinical application. Infusionstherapie. 1991;18(6):271-8.

36. Bellisle F. Sugar-sweetened drinks and body weight control. Cahiers de Nutrition et de Dietetique. 2011;46(1 SUPPL. 1):S20-S6.

37. Bellisle F, Blundell JE, Dye L, Fantino M, Fern E, Fletcher RJ, et al. Functional food science and behaviour and psychological functions. British Journal of Nutrition. 1998;80:S173-S93. PubMed PMID: WOS:000076160300008.

38. Bellisle F, Drewnowski A, Anderson GH, Westerterp-Plantenga M, Martin CK. Sweetness, satiation, and satiety. Journal of Nutrition. 2012;142(6):1149S-54S.

39. Belza A, Larsen BE, Pedersen SB, Astrup A, Richelsen B. Indication of more satiating effect of milk compared to isocaloric sucrose-sweetened soft drink Nielsen MM. Obesity Reviews. 2010;11:438-9.

40. Bender DA. Nutrition Research Reviews: Editorial. Nutrition Research Reviews. 2005;18(1):1-2.

41. Benoit SC, Davis JF, Davidson TL. Learned and cognitive controls of food intake. Brain Res. 2010;1350:71-6. Epub 2010/06/22. doi: 10.1016/j.brainres.2010.06.009. PubMed PMID: 20561510; PubMed Central PMCID: PMC2926208.

42. Bilton R. Averting comfortable lifestyle crises. Science progress. 2013;96(Pt 4):319-68. Epub 2014/02/20. PubMed PMID: 24547668.

43. Bleich SN, Wolfson JA, Vine S, Wang YC. Diet-beverage consumption and caloric intake among US adults, overall and by body weight. American Journal of Public Health. 2014;104(3):e72-e8.

44. Blundell JE, Rogers PJ, Hill AJ. Uncoupling sweetness and calories: methodological aspects of laboratory studies on appetite control. Appetite. 1988;11 Suppl 1:54-61. Epub 1988/01/01. PubMed PMID: 3056268.

45. Bogdanov S, Jurendic T, Sieber R, Gallmann P. Honey for nutrition and health: A review. Journal of the American College of Nutrition. 2008;27(6):677-89.

46. Borys JM, De Ruyter JC, Finch H, Harper P, Levy E, Mayer J, et al. Hydration and obesity prevention. Obesity Facts. 2014;7(SUPPL. 2):37-48.

47. Boulos R, Vikre EK, Oppenheimer S, Chang H, Kanarek RB. ObesiTV: How television is influencing the obesity epidemic. Physiology and Behavior. 2012;107(1):146-53.

48. Bourne LT, Harmse B, Temple N. Water: a neglected nutrient in the young child? A South African perspective. Maternal & child nutrition. 2007;3(4):303-11. Epub 2007/09/11. doi: 10.1111/j.1740-8709.2007.00114.x. PubMed PMID: 17824858.

49. Boyet F. Do's and don'ts with a woman taking the oral contraceptive pill and with an abnormal lipid pattern. Contraception Fertilite Sexualite. 1984;12(9):1076-7.

50. Brahmini M, Keerthi T, Priyadarshini B, Sudheerbabu I. Myths and facts about aspartame and sucralose: A critical review. International Journal of Research in Ayurveda and Pharmacy. 2012;3(3):373-5.

51. Bray GA. The epidemic of obesity and changes in food intake: the Fluoride Hypothesis. Physiology & behavior. 2004;82(1):115-21. Epub 2004/07/06. doi: 10.1016/j.physbeh.2004.04.033. PubMed PMID: 15234599.

52. Bray GA. Fructose: Should we worry? International Journal of Obesity. 2008;32(SUPPL. 7):S127-S31.

53. Bray GA. Are non-prescription medications needed for weight control? Obesity. 2008;16(3):509-14.

54. Bray GA. Fructose and risk of cardiometabolic disease. Curr Atheroscler Rep. 2012;14(6):570-8. Epub 2012/09/06. doi: 10.1007/s11883-012-0276-6. PubMed PMID: 22949106.

55. Bray GA. Energy and fructose from beverages sweetened with sugar or high-fructose corn syrup pose a health risk for some people. Advances in Nutrition. 2013;4(2):220-5.

56. Bray GA, Nielsen SJ, Popkin BM. Consumption of high-fructose corn syrup in beverages may play a role in the epidemic of obesity. American Journal of Clinical Nutrition. 2004;79(4):537-43.

57. Bray GA, Popkin BM. Epidemics of obesity and metabolic disorders: Are dietary fats or sugars involved? Current Nutrition and Food Science. 2007;3(2):113-21.

58. Bray GA, Popkin BM. Calorie-sweetened beverages and fructose: What have we learned 10 years later. Pediatric Obesity. 2013;8(4):242-8.

59. Bray GA, Popkin BM. Dietary Sugar and Body Weight: Have We Reached a Crisis in the Epidemic of Obesity and Diabetes? Diabetes Care. 2014;37(4):950-6. doi: 10.2337/dc13-2085. PubMed PMID: WOS:000333414700023.

60. Bremer S, Hartung T. The use of embryonic stem cells for regulatory developmental toxicity testing in vivo - The current status of test development. Current Pharmaceutical Design. 2004;10(22):2733-47.

61. Briefel RR, Crepinsek MK, Cabili C, Wilson A, Gleason PM. School Food Environments and Practices Affect Dietary Behaviors of US Public School Children. Journal of the American Dietetic Association. 2009;109(2):S91-S107.

62. Briffa J, Gordon IJ, Finer N, Lean MEJ, Hankey CR. Aspartame and its effects on health [5] (multiple letters). British Medical Journal. 2005;330(7486):309-10.

63. Brown CM, Dulloo AG, Montani JP. Sugary drinks in the pathogenesis of obesity and cardiovascular diseases. International Journal of Obesity. 2008;32(SUPPL. 6):S28-S34.

64. Brunzell JD. Use of fructose, sorbitol, or xylitol as a sweetener in diabetes mellitus. Journal of the American Dietetic Association. 1978;73(5):499-506.

65. Brunzell JD. Use of Fructose, Sorbitol, or Xylitol as a Sweetener in Diabetes-Mellitus. Journal of the American Dietetic Association. 1978;73(5):499-506. PubMed PMID: WOS:A1978FV28200001.

66. Brusick DJ. A critical review of the genetic toxicity of steviol and steviol glycosides. Food and Chemical Toxicology. 2008;46(7 SUPPL.):S83-S91.

67. Burke LM, Castell LM, Stear SJ, Rogers PJ, Blomstrand E, Gurr S, et al. BJSM reviews: A-Z of nutritional supplements: dietary supplements, sports nutrition foods and ergogenic aids for health and performance Part 4. Br J Sports Med. 2009;43(14):1088-90. Epub 2009/12/04. doi: 10.1136/bjsm.2009.068643. PubMed PMID: 19955167.

68. Butchko HH, Stargel WW. Aspartame: Scientific evaluation in the postmarketing period. Regulatory Toxicology and Pharmacology. 2001;34(3):221-33.

69. Butchko HH, Stargel WW, Comer CP, Mayhew DA, Benninger C, Blackburn GL, et al. Aspartame: review of safety. Regulatory Toxicology and Pharmacology. 2002;35(2 Pt 2):S1-93.

70. Butchko HH, Stargel WW, Comer CP, Mayhew DA, Benninger C, Blackburn GL, et al. Regulatory toxicology and pharmacology. Regulatory Toxicology and Pharmacology. 2002;35(2 II):S1-S93.

71. Cabanillas M, Moya Chimenti E, Gonzalez Candela C, Loria Kohen V, Dassen C, Lajo T. [Usefulness of meal replacement: analysis of the principal meal replacement products commercialised in Spain]. Nutr Hosp. 2009;24(5):535-42. Epub 2009/11/07. PubMed PMID: 19893863.

72. Cabrera Escobar MA, Veerman JL, Tollman SM, Bertram MY, Hofman KJ. Evidence that a tax on sugar sweetened beverages reduces the obesity rate: A meta-analysis. BMC Public Health. 2013;13(1).

73. Cameron A, Thornton L. The availability of energy-dense snack foods, soft drinks, fruit and vegetables in urban, urban-fringe and rural supermarkets. Obesity Reviews. 2014;15:191.

74. Capeau J. Insulin signaling: Mechanisms altered in insulin resistance. Voies de signalisation de l'insuline: Mécanismes affectés dans l'insulino-résistance. 2003;19(8-9):834-9.

75. Carvalhana S, Machado MV, Cortez-Pinto H. Improving dietary patterns in patients with nonalcoholic fatty liver disease. Current Opinion in Clinical Nutrition and Metabolic Care. 2012;15(5):468-73. doi: 10.1097/MCO.0b013e3283566614. PubMed PMID: WOS:000307826500011.

76. Chan TYT. Dietary management of type 2 diabetes mellitus. Hong Kong Practitioner. 2003;25(1):22-8.

77. Chen L, Hu FB, Yeung E, Willett W, Zhang C. Prospective study of pre-gravid sugar-sweetened beverage consumption and the risk of gestational diabetes mellitus. Diabetes Care. 2009;32(12):2236-41.

78. Chong MFF, Fielding BA, Frayn KN. Metabolic interaction of dietary sugars and plasma lipids with a focus on mechanisms and de novo lipogenesis. Proceedings of the Nutrition Society. 2007;66(1):52-9.

79. Choudhary P. Review of dietary recommendations for diabetes mellitus. Diabetes Res Clin Pract. 2004;65 Suppl 1(SUPPL.):S9-S15. Epub 2004/08/19. doi: 10.1016/j.diabres.2004.07.003. PubMed PMID: 15315865.

80. Chung MY, Oh DK, Lee KW. Hypoglycemic health benefits of D-psicose. J Agric Food Chem. 2012;60(4):863-9. Epub 2012/01/10. doi: 10.1021/jf204050w. PubMed PMID: 22224918.

81. Clabaugh K, Neuberger GB. Research evidence for reducing sugar sweetened beverages in children. Issues in Comprehensive Pediatric Nursing. 2011;34(3):119-30.

82. Coade G, Holly JMP, Shield JPH, Welsh GI, Foulstone EJ. Human visceral preadipocytes have a greater capacity to transport fructose compared to subcutaneous preadipocytes. Endocrine reviews. 2011;32(3).

83. Cohen DA, Bhatia R. Nutrition standards for away-from-home foods in the USA. Obesity Reviews. 2012;13(7):618-29.

84. Colberg SR, Sigal RJ, Fernhall B, Regensteiner JG, Blissmer BJ, Rubin RR, et al. Exercise and Type 2 Diabetes The American College of Sports Medicine and the American Diabetes Association joint position statement executive summary. Diabetes Care. 2010;33(12):2692-6. doi: Doi 10.2337/Dc10.1548. PubMed PMID: WOS:000285666200039.

85. Connor H. Sucrose and fructose in the diabetic diet. Journal of Human Nutrition and Dietetics. 1991;4(4):243-50.

86. Corkey BE. Diabetes: Have we got it all wrong? Insulin hypersecretion and food additives: Cause of obesity and diabetes? Diabetes Care. 2012;35(12):2432-7.

87. Costa FV. Non-pharmacological treatment of hypertension in women. Journal of Hypertension. 2002;20:S57-S61. PubMed PMID: WOS:000176186100014.

88. Cozma AI, Sievenpiper JL. The role of fructose, sucrose, and high-fructose corn syrup in diabetes. European Endocrinology. 2013;9(2).

89. Cradock AL, McHugh A, Mont-Ferguson H, Grant L, Barrett JL, Wang YC, et al. Effect of school district policy change on consumption of sugar-sweetened beverages among high school students, Boston, Massachusetts, 2004-2006. Preventing Chronic Disease. 2011;8(4).

90. Crowe JH, Crowe LM, Carpenter JF, Rudolph AS, Wistrom CA, Spargo BJ, et al. Interactions of sugar with membranes. Biochimica et Biophysica Acta - Reviews on Biomembranes. 1988;947(2):367-84.

91. Cui M, Jiang PH, Maillet E, Max M, Margolskee RF, Osman R. The heterodimeric sweet taste receptor has multiple potential ligand binding sites. Current Pharmaceutical Design. 2006;12(35):4591-600. doi: Doi 10.2174/138161206779010350. PubMed PMID: WOS:000242919900006.

92. Dallman MF, Akana SF, Pecoraro NC, Warne JP, la Fleur SE, Foster MT. Glucocorticoids, the etiology of obesity and the metabolic syndrome. Curr Alzheimer Res. 2007;4(2):199-204. Epub 2007/04/14. PubMed PMID: 17430247.

93. Dandona P, Chaudhuri A, Ghanim H, Mohanty P. Proinflammatory effects of glucose and anti-inflammatory effect of insulin: relevance to cardiovascular disease. The American journal of cardiology. 2007;99(4a):15b-26b. Epub 2007/02/20. doi: 10.1016/j.amjcard.2006.11.003. PubMed PMID: 17307055.

94. Daudon M. [Epidemiology of nephrolithiasis in France]. Ann Urol (Paris). 2005;39(6):209-31. Epub 2006/01/24. PubMed PMID: 16425740.

95. Davidson GS. Including pets in diabetic care. International Journal of Pharmaceutical Compounding. 2007;11(2):124-8.

96. Davidson TL, Sample CH, Swithers SE. An application of Pavlovian principles to the problems of obesity and cognitive decline. Neurobiology of Learning and Memory. 2014;108:172-84.

97. De Ruyter JC, Olthof MR, Katan MB. Rationale and design of the double-blind, randomized intervention study in kids (DRINK) on the effect of sugary drinks on body weight and fat mass. Obesity Reviews. 2010;11:190-1.

98. Debry G, Drouin P, Gariot P, Pointel JP, Louis J, Gross P, et al. Nutritional management of diabetes mellitus: Rationale, ethics and practicability. Journal of the American College of Nutrition. 1986;5(1):9-30.

99. Dehghan M, Akhtar-Danesh N, Merchant AT. Childhood obesity, prevalence and prevention. Nutrition Journal. 2005;4.

100. Derosa G, Limas CP, Maciás PC, Estrella A, Maffioli P. Dietary and nutraceutical approach to type 2 diabetes. Archives of Medical Science. 2014;10(2):336-44.

101. Dotson CD, Babich J, Steinle NI. Genetic Predisposition and Taste Preference: Impact on Food Intake and Risk of Chronic Disease. Current Nutrition Reports. 2012;1(3):175-83.

102. Drewnowski A. The real contribution of added sugars and fats to obesity. Epidemiologic Reviews. 2007;29(1):160-71.

103. Drewnowski A. Low-calorie sweeteners in the management of obesity and diabetes. Diabetes, Obesity and Metabolism. 2010;12:33-4.

104. Drewnowski A, Almiron-Roig E, Marmonier C, Lluch A. Dietary energy density and body weight: is there a relationship? Nutr Rev. 2004;62(11):403-13. Epub 2004/12/30. PubMed PMID: 15622713.

105. Drewnowski A, Popkin BM. The nutrition transition: New trends in the global diet. Nutrition Reviews. 1997;55(2):31-43.

106. Edwards RD. Commentary: Soda taxes, obesity, and the shifty behavior of consumers. Preventive Medicine. 2011;52(6):417-8. doi: 10.1016/j.ypmed.2011.04.011. PubMed PMID: WOS:000291758700003.

107. Elliott SS, Keim NL, Stern JS, Teff K, Havel PJ. Fructose, weight gain, and the insulin resistance syndrome. The American journal of clinical nutrition. 2002;76(5):911-22. Epub 2002/10/26. PubMed PMID: 12399260.

108. Ensinck JW, Bierman EL. Dietary management of diabetes mellitus. Annual Review of Medicine. 1979;30:155-70.

109. Erejuwa OO, Sulaiman SA, Ab Wahab MS. Fructose Might Contribute to the Hypoglycemic Effect of Honey. Molecules. 2012;17(2):1900-15. doi: 10.3390/molecules17021900. PubMed PMID: WOS:000300717200053.

110. Erlanson-Albertsson C. Appetite regulation and energy balance. Acta paediatrica (Oslo, Norway : 1992) Supplement. 2005;94(448):40-1. Epub 2005/09/24. PubMed PMID: 16175806.

111. Ernest TB, Elder DP, Martini LG, Roberts M, Ford JL. Developing paediatric medicines: identifying the needs and recognizing the challenges. The Journal of pharmacy and pharmacology. 2007;59(8):1043-55. Epub 2007/08/30. doi: 10.1211/jpp.59.8.0001. PubMed PMID: 17725846.

112. Evert AB, Boucher JL, Cypress M, Dunbar SA, Franz MJ, Mayer-Davis EJ, et al. Nutrition therapy recommendations for the management of adults with diabetes. Diabetes Care. 2014;37(SUPPL.1):S120-S43.

113. Eyles H, Mhurchu CN, Nghiem N, Blakely T. Food Pricing Strategies, Population Diets, and Non-Communicable Disease: A Systematic Review of Simulation Studies. Plos Medicine. 2012;9(12). doi: 10.1371/journal.pmed.1001353. PubMed PMID: WOS:000312934200003.

114. Ezhumalai K, Ilavarasan P, Rajalakshmi AN, Sathiyaraj U, Murali Mugundhan R. Medicated chewing gum-a novel drug delivery technique for systemic and targeted drug delivery. International Journal of Pharmacy and Technology. 2011;3(1):725-44.

115. Faus I. Recent developments in the characterization and biotechnological production of sweet-tasting proteins. Appl Microbiol Biotechnol. 2000;53(2):145-51. Epub 2000/03/10. PubMed PMID: 10709975.

116. Ferder L, Ferder MD, Inserra F. The role of high-fructose corn syrup in metabolic syndrome and hypertension. Current Hypertension Reports. 2010;12(2):105-12.

117. Feron VJ, van Vliet PW, Notten WR. Exposure to combinations of substances: a system for assessing health risks. Environ Toxicol Pharmacol. 2004;18(3):215-22. Epub 2004/12/01. doi: 10.1016/j.etap.2003.11.009. PubMed PMID: 21782751.

118. Ferreira AV, Generoso SV, Teixeira AL. Do low-calorie drinks 'cheat' the enteral-brain axis? Curr Opin Clin Nutr Metab Care. 2014;17(5):465-70. Epub 2014/07/16. doi: 10.1097/mco.0000000000000082. PubMed PMID: 25023189.

119. Ferris i Tortajada J, Lopez Andreu JA, Benedito Monleon MC, Garcia i Castell J. [The pediatrician and cancer prevention. Dietetic factors and smoking]. An Esp Pediatr. 1996;45(1):6-13. Epub 1996/07/01. PubMed PMID: 8849133.

120. Ferrís i Tortajada J, López Andreu JA, Benedito Monleón MC, García i Castell J. Pediatrics and oncological prevention: Factors related to diet and tobacco. El pediatria y la prevencion oncologica Factores dieteticos y tabaquismo. 1996;45(1):6-13.

121. Fidler Mis N. Negative effects of sugar-sweetened beverages. Zdravniski Vestnik. 2013;82(SUPPL.1):I138-I44.

122. Filer LJ, Jr., Stegink LD. Aspartame metabolism in normal adults, phenylketonuric heterozygotes, and diabetic subjects. Diabetes Care. 1989;12(1):67-74. Epub 1989/01/01. PubMed PMID: 2653751.

123. Foerster H, Haslbeck M, Mehnert H. Carbohydrates in parenteral nutrition. ZUR BEDEUTUNG DER KOHLENHYDRATE IN DER PARENTERALEN ERNAHRUNG. 1974;1(3):199-213.

124. Fonfa AE. Patient perspectives: Barriers to complementary and alternative medicine therapies create problems for patients and survivors. Integrative Cancer Therapies. 2007;6(3):297-300.

125. Forshee RA, Storey ML, Allison DB, Glinsmann WH, Hein GL, Lineback DR, et al. A critical examination of the evidence relating high fructose corn syrup and weight gain. Crit Rev Food Sci Nutr. 2007;47(6):561-82. Epub 2007/07/27. doi: 10.1080/10408390600846457. PubMed PMID: 17653981.

126. Forster H. Influence of the sweetening agent aspartame on appetite. EINFLUSS DES SUSSSTOFFS ASPARTAME AUF DEN APPETIT. 1993;18(5):331-7.

127. Fortuna JL. Sweet preference, sugar addiction and the familial history of alcohol dependence: Shared neural pathways and genes. Journal of Psychoactive Drugs. 2010;42(2):147-51.

128. Franz MJ, Bantle J, Beebe CA, Brunzell JD, Chiasson JL, Garg A, et al. American Diabetes Association Position Statement: Evidence-based nutrition principles and recommendations for the treatment and prevention of diabetes and related complications. Journal of the American Dietetic Association. 2002;102(1):109-18.

129. Franz MJ, Bantle JP, Beebe CA, Brunzell JD, Chiasson JL, Garg A, et al. Nutrition principles and recommendations in diabetes. Diabetes Care. 2004;27 Suppl 1(SUPPL. 1):S36-46. Epub 2003/12/25. PubMed PMID: 14693924.

130. Franz MJ, Horton ES, Bantle JP, Beebe CA, Brunzell JD, Coulston AM, et al. Nutrition principles for the management of diabetes and related complications. Diabetes Care. 1994;17(5):490-518.

131. Fung A, Vizcaychipi M, Lloyd D, Wan Y, Ma D. Central nervous system inflammation in disease related conditions: mechanistic prospects. Brain Res. 2012;1446:144-55. Epub 2012/02/22. doi: 10.1016/j.brainres.2012.01.061. PubMed PMID: 22342162.

132. Fung M, Tildesley H, Gill S. New treatments and treatment philosophy for type 1 diabetes. British Columbia Medical Journal. 2004;46(9):451-6.

133. Gaby AR. Adverse effects of dietary fructose. Alternative Medicine Review. 2005;10(4):294-306.

134. Gallagher EJ, LeRoith D. Insulin, insulin resistance, obesity, and cancer. Curr Diab Rep. 2010;10(2):93-100. Epub 2010/04/29. doi: 10.1007/s11892-010-0101-y. PubMed PMID: 20425567.

135. Geuns JM. Stevioside. Phytochemistry. 2003;64(5):913-21. Epub 2003/10/17. PubMed PMID: 14561506.

136. Gibbs BF, Alli I, Mulligan C. Sweet and taste-modifying proteins: A review. Nutrition Research. 1996;16(9):1619-30.

137. Gibson EL. The psychobiology of comfort eating: Implications for neuropharmacological interventions. Behavioural Pharmacology. 2012;23(5-6):442-60.

138. Gilden JL. Nutrition and the older diabetic. Clin Geriatr Med. 1999;15(2):371-90. Epub 1999/05/26. PubMed PMID: 10339639.

139. Glinsmann WH, Irausquin H, Park YK. Evaluation of health aspects of sugars contained in carbohydrate sweeteners. Report of Sugar Task Force, 1986. Journal of Nutrition. 1986;116(11 SUPPL.):S1-S216.

140. Goldey ES, Tilson HA, Crofton KM. Implications of the use of neonatal birth weight, growth, viability, and survival data for predicting developmental neurotoxicity: A survey of the literature. Neurotoxicology and Teratology. 1995;17(3):313-32.

141. Golub M, Costa L, Crofton K, Frank D, Fried P, Gladen B, et al. NTP-CERHR Expert Panel Report on the reproductive and developmental toxicity of methylphenidate. Birth defects research Part B, Developmental and reproductive toxicology. 2005;74(4):300-81. Epub 2005/08/30. doi: 10.1002/bdrb.20049. PubMed PMID: 16127684.

142. Gould M, Sellin JH. Diabetic diarrhea. Current Gastroenterology Reports. 2009;11(5):354-9.

143. Gregory JM, Moore DJ, Simmons JH. Type 1 diabetes mellitus. Pediatrics in Review. 2013;34(5):203-15.

144. Grillaud M, Bandon D, Nancy J, Delbos Y, Vaysse F. [The polyols in pediatric dentistry: advantages of xylitol]. Archives de pediatrie : organe officiel de la Societe francaise de pediatrie. 2005;12(7):1180-6. Epub 2005/06/21. doi: 10.1016/j.arcped.2005.01.032. PubMed PMID: 15964535.

145. Grosjean OV. The resistible growth of health care costs. Acta Chirurgica Belgica. 2008;108(1):4-14.

146. Grotz VL, Kruger CL, Flora G, Hayes AW. Mutagenicity review of sucralose, a popular no-calorie sweetener. International Journal of Toxicology. 2009;28(1):57.

147. Grotz VL, Munro IC. An overview of the safety of sucralose. Regulatory Toxicology and Pharmacology. 2009;55(1):1-5.

148. Guay DRP. Cranberry and urinary tract infections. Drugs. 2009;69(7):775-807.

149. Gwynn J. The impact of an Australian indigenous community governed program of health promotion on key risk factors for obesity: The results of the many rivers diabetes prevention project. Obesity Reviews. 2014;15:213.

150. Hafekost K, Mitrou F, Lawrence D, Zubrick SR. Sugar sweetened beverage consumption by Australian children: Implications for public health strategy. BMC Public Health. 2011;11.

151. Haghighi M, Rezaei K. Designing an all-apple-pomace-based functional dessert formulation. British Food Journal. 2013;115(3):409-24.

152. Halsted CH. Perspectives on obesity and sweeteners, folic acid fortification and vitamin D requirements. Family Practice. 2009;25(SUPPL. 1):i44-i9.

153. Hankey GJ. Nutrition and the risk of stroke. The Lancet Neurology. 2012;11(1):66-81.

154. Harrison D, Bueno M. Sweet solutions for pain in infants - How many studies are too many studies. Pain Research and Management. 2012;17(3):211.

155. Hashemi Kani A, Alavian SM, Haghighatdoost F, Azadbakht L. Diet macronutrients composition in nonalcoholic fatty liver disease: A review on the related documents. Hepatitis Monthly. 2014;14(2).

156. Haslbeck M. Nutrition and diseases - The role of carbohydrates. Wiener Medizinische Wochenschrift. 1990;140(SUPPL. 106):7-10.

157. Haslbeck M. [Diet and civilization diseases--carbohydrate aspects]. Wiener medizinische Wochenschrift Supplement. 1990;106:suppl 7-10. Epub 1990/01/01. PubMed PMID: 2171232.

158. Hauner H. Consumption of confectionery products from the diabetologist's point of view. Aktuelle Ernahrungsmedizin. 1999;24(SUPPL.):66-72.

159. Havermans RC. Pavlovian Craving and Overeating: A Conditioned Incentive Model. Current Obesity Reports. 2013;2(2):165-70.

160. Hawkes C. Uneven dietary development: Linking the policies and processes of globalization with the nutrition transition, obesity and diet-related chronic diseases. Globalization and Health. 2006;2.

161. Hawkes C. The Worldwide Battle Against Soft Drinks in Schools. American Journal of Preventive Medicine. 2010;38(4):457-61.

162. Heilberg IP, Goldfarb DS. Optimum Nutrition for Kidney Stone Disease. Advances in Chronic Kidney Disease. 2013;20(2):165-74.

163. Hensrud DD. Diet and obesity. Current Opinion in Gastroenterology. 2004;20(2):119-24.

164. Hodgson J. Obesity: Where less is more. Bio/Technology. 1995;13(10):1060-3.

165. Hodgson J. Obesity - Where Less Is More. Bio-Technol. 1995;13(10):1060-3. doi: Doi 10.1038/Nbt1095-1060. PubMed PMID: WOS:A1995RY31800020.

166. Horio N, Jyotaki M, Yoshida R, Sanematsu K, Shigemura N, Ninomiya Y. New frontiers in gut nutrient sensor research: nutrient sensors in the gastrointestinal tract: modulation of sweet taste sensitivity by leptin. Journal of pharmacological sciences. 2010;112(1):8-12. Epub 2010/01/23. PubMed PMID: 20093782.

167. Imamura M, Williams K, Wells M, McGrother C. Clinical effectiveness of lifestyle interventions for adults with urinary incontinence: A systematic review of randomised trials. Neurourology and Urodynamics. 2013;32(6):656.

168. Ines Mussatto S, Roberto Conceicao I. Xylitol: A sweetner with benefits for human health. Revista Brasileira de Ciencias Farmaceuticas/Brazilian Journal of Pharmaceutical Sciences. 2002;38(4):401-13.

169. Ingole BD, Daga AS, Joshi UM, Biyani KR. Chewing gum: A mobile drug delivery system. International Journal of Pharmaceutical Sciences Review and Research. 2012;14(2):106-14.

170. Jardine NJ, Edwards DG, McMenemy C. Implications of nutritional recommendations on sugar for product development. Proceedings of the Nutrition Society. 1990;49(1):23-30.

171. Jia G, Aroor AR, Whaley-Connell AT, Sowers JR. Fructose and uric acid: Is there a role in endothelial function? Current Hypertension Reports. 2014;16(6).

172. Johnson L, Wilks DC, Lindroos AK, Jebb SA. Reflections from a systematic review of dietary energy density and weight gain: is the inclusion of drinks valid? Obesity Reviews. 2009;10(6):681-92. doi: 10.1111/j.1467-789X.2009.00580.x. PubMed PMID: WOS:000271250600011.

173. Johnson RJ, Nakagawa T, Sanchez-Lozada LG, Shafiu M, Sundaram S, Le M, et al. Sugar, uric acid, and the etiology of diabetes and obesity. Diabetes. 2013;62(10):3307-15. Epub 2013/09/26. doi: 10.2337/db12-1814. PubMed PMID: 24065788; PubMed Central PMCID: PMCPmc3781481.

174. Johnson RJ, Perez-Pozo SE, Sautin YY, Manitius J, Sanchez-Lozada LG, Feig DI, et al. Hypothesis: Could excessive fructose intake and uric acid cause type 2 diabetes? Endocrine reviews. 2009;30(1):96-116.

175. Johnson RJ, Segal MS, Sautin Y, Nakagawa T, Feig DI, Kang DH, et al. Potential role of sugar (fructose) in the epidemic of hypertension, obesity and the metabolic syndrome, diabetes, kidney disease, and cardiovascular disease1-3. American Journal of Clinical Nutrition. 2007;86(4):899-906.

176. Johnson RK, Frary C. Choose beverages and foods to moderate your intake of sugars: the 2000 dietary guidelines for Americans--what's all the fuss about? The Journal of nutrition. 2001;131(10):2766s-71s. Epub 2001/10/05. PubMed PMID: 11584103.

177. Jones HF, Burt E, Metcalfe M, Dowling K, Davidson G, Brooks DA, et al. Fructose malabsorption from infancy to adulthood in patients with gastrointestinal symptoms. Gastroenterology. 2010;138(5):S586.

178. Justel N, Bentosela M, Mustaca A, Ruetti E. Neonatal treatment with clomipramine and depression: A review of behavioral and physiological findings. Interdisciplinaria. 2012;28(2):207-20.

179. Kabra AO, Shah FB, Wanare RS. Formulation and in vitro evaluation of rapidly disintegrating tablets using captopril as a model drug. International Journal of Pharmaceutical Sciences Review and Research. 2011;7(2):206-10.

180. Kahn R, Sievenpiper JL. Dietary Sugar and Body Weight: Have We Reached a Crisis in the Epidemic of Obesity and Diabetes? Diabetes Care. 2014;37(4):957-62. doi: 10.2337/dc13-2506. PubMed PMID: WOS:000333414700024.

181. Kant R. Sweet proteins--potential replacement for artificial low calorie sweeteners. Nutr J. 2005;4:5. doi: 10.1186/1475-2891-4-5. PubMed PMID: 15703077; PubMed Central PMCID: PMC549512.

182. Karalius VP, Shoham DA. Dietary sugar and artificial sweetener intake and chronic kidney disease: a review. Adv Chronic Kidney Dis. 2013;20(2):157-64. Epub 2013/02/27. doi: 10.1053/j.ackd.2012.12.005. PubMed PMID: 23439375.

183. Kathpalia H, Gupte A. An introduction to fast dissolving oral thin film drug delivery systems: A review. Current Drug Delivery. 2013;10(6):667-84.

184. Kaur H, Hyder ML, Poston WSC. Childhood Overweight: An Expanding Problem. Treatments in Endocrinology. 2003;2(6):375-88.

185. Kellett GL, Brot-Laroche E, Mace OJ, Leturque A. Sugar absorption in the intestine: The role of GLUT2. 2008. p. 35-54.

186. Kelley AE, Bakshi VP, Haber SN, Steininger TL, Will MJ, Zhang M. Opioid modulation of taste hedonics within the ventral striatum. Physiology and Behavior. 2002;76(3):365-77.

187. Kelly C, Molcho M, Nic Gabhainn S. Patterns in weight reduction behaviour by weight status in schoolchildren. Public Health Nutrition. 2010;13(8):1229-36.

188. Khanekar P, Mhatre S, Momin M. Medicated chewing gum: A potential drug delivery system. International Journal of Pharmaceutical Frontier Research. 2012;2(4):64-75.

189. King RA. The role of polyphenols in human health. Brooker JD, editor2000. 75-81 p.

190. Klurfeld DM. What do government agencies consider in the debate over added sugars? Advances in Nutrition. 2013;4(2):257-61.

191. Ko BJ, Park KH, Mantzoros CS. Diet patterns, adipokines, and metabolism: Where are we and what is next? Metabolism: clinical and experimental. 2014;63(2):168-77.

192. Kroger M, Meister K, Kava R. Low-calorie sweeteners and other sugar substitutes: A review of the safety issues. Comprehensive Reviews in Food Science and Food Safety. 2006;5(2):35-47. doi: 10.1111/j.1541-4337.2006.tb00081.x. PubMed PMID: WOS:000244468700002.

193. Kumanyika S, Taylor WC, Grier SA, Lassiter V, Lancaster KJ, Morssink CB, et al. Community energy balance: A framework for contextualizing cultural influences on high risk of obesity in ethnic minority populations. Preventive Medicine. 2012;55(5):371-81. doi: 10.1016/j.ypmed.2012.07.002. PubMed PMID: WOS:000311061100004.

194. Kumar A, Saroha K, Mohan R, Chetna K. A review on sublingual tablets. Pharma Research. 2012;8(1):98-111.

195. Kurihara K, Suzuki T, Unno A, Hatano M. [Case of 5 year-old boy with anaphylaxis due to erythritol with negative prick test and positive intradermal test]. Arerugī = [Allergy]. 2013;62(11):1534-40.

196. Kushi LH, Byers T, Doyle C, Bandera EV, McCullough M, McTiernan A, et al. American Cancer Society Guidelines on Nutrition and Physical Activity for cancer prevention: reducing the risk of cancer with healthy food choices and physical activity. CA: a cancer journal for clinicians. 2006;56(5):254-81; quiz 313-4. Epub 2006/09/29. PubMed PMID: 17005596.

197. Lacroix C, Payne A, Chassard C. Fructose impacts on gut microbiota and obesity - response to H. C. Stevens. Obesity Reviews. 2012;13(12):1184-5.

198. Laffitte A, Neiers F, Briand L. Functional roles of the sweet taste receptor in oral and extraoral tissues. Current Opinion in Clinical Nutrition and Metabolic Care. 2014;17(4):379-85. doi: 10.1097/mco.0000000000000058. PubMed PMID: WOS:000337282500014.

199. Lairon D. Postprandial lipid metabolism and its pathophysiological consequences. Lipémie postprandiale: Réponses aux nutriments et conséquences physio-pathologiques. 2008;43(4):186-90.

200. Lakhan SE, Kirchgessner A. The emerging role of dietary fructose in obesity and cognitive decline. Nutrition Journal. 2013;12(1).

201. Lasater G, Piernas C, Popkin BM. Beverage patterns and trends among school-aged children in the US, 1989-2008. Nutrition Journal. 2011;10(1).

202. Lavin PT, Sanders PG, Mackey MA, Kotsonis FN. Intense sweeteners use and weight change among women: A critique of the Stellman and Garfinkel study. Journal of the American College of Nutrition. 1994;13(1):102-5.

203. Lawrence GD. Dietary fats and health: Dietary recommendations in the context of scientific evidence. Advances in Nutrition. 2013;4(3):294-302.

204. Leclercq IA, Horsmans Y. Nonalcoholic fatty liver disease: the potential role of nutritional management. Current Opinion in Clinical Nutrition and Metabolic Care. 2008;11(6):766-73. doi: 10.1097/MCO.0b013e328312c353. PubMed PMID: WOS:000260619600014.

205. Lee C, Longo VD. Fasting vs dietary restriction in cellular protection and cancer treatment: from model organisms to patients. Oncogene. 2011;30(30):3305-16. Epub 2011/04/26. doi: 10.1038/onc.2011.91. PubMed PMID: 21516129.

206. Lee S, Kim Y. Effects of exercise alone on insulin sensitivity and glucose tolerance in obese youth. Diabetes and Metabolism Journal. 2013;37(4):225-32.

207. Levine AS, Kotz CM, Gosnell BA. Sugars: hedonic aspects, neuroregulation, and energy balance. American Journal of Clinical Nutrition. 2003;78(4):834S-42S. PubMed PMID: WOS:000185403700028.

208. Levine R. Monosaccharides in health and disease. Annu Rev Nutr. 1986;6:211-24. Epub 1986/01/01. doi: 10.1146/annurev.nu.06.070186.001235. PubMed PMID: 3524617.

209. Levy DT, Friend KB. Simulation Modeling of Policies Directed at Youth Sugar-Sweetened Beverage Consumption. American Journal of Community Psychology. 2013;51(1-2):299-313. doi: 10.1007/s10464-012-9535-5. PubMed PMID: WOS:000314834100026.

210. Li X, Alexander K. Proteins used as sweeteners: A review. International Journal of Pharmaceutical Compounding. 2007;11(6):476-80.

211. Libuda L, Kersting M. Soft drinks and body weight development in childhood: is there a relationship? Curr Opin Clin Nutr Metab Care. 2009;12(6):596-600. Epub 2009/08/28. doi: 10.1097/MCO.0b013e32833189f6. PubMed PMID: 19710610.

212. Lina BAR, Jonker D, Kozianowski G. Isomaltulose (Palatinose®): A review of biological and toxicological studies. Food and Chemical Toxicology. 2002;40(10):1375-81.

213. Livesey G. Health potential of polyols as sugar replacers, with emphasis on low glycaemic properties. Nutrition Research Reviews. 2003;16(2):163-91.

214. Livesey G. Fructose, Obesity, and Related Epidemiology. Critical Reviews in Food Science and Nutrition. 2010;50:26-8. doi: 10.1080/10408398.2010.526870. PubMed PMID: WOS:000284955300010.

215. Livesey G, Taylor R. Fructose consumption and consequences for glycation, plasma triacylglycerol, and body weight: Meta-analyses and meta-regression models of intervention studies. American Journal of Clinical Nutrition. 2008;88(5):1419-37.

216. Lombardi VRM, Lombardi C, Cacabelos R. Nutrition, immune system activation and anti-cancer strategies in animals and humans. Current Topics in Pharmacology. 2008;12(2):1-30.

217. Lottenberg AM. [Diet composition along the evolution of type 1 diabetes mellitus]. Arq Bras Endocrinol Metabol. 2008;52(2):250-9. Epub 2008/04/29. PubMed PMID: 18438535.

218. Lottenberg AMP. Diet composition along the evolution of Type 1 diabetes mellitus. Arquivos Brasileiros de Endocrinologia e Metabologia. 2008;52(2):250-9.

219. Lu Y, Levin GV, Donner TW. Tagatose, a new antidiabetic and obesity control drug. Diabetes, obesity & metabolism. 2008;10(2):109-34. Epub 2007/10/19. doi: 10.1111/j.1463-1326.2007.00799.x. PubMed PMID: 17941870.

220. Lucas SD, Costa E, Guedes RC, Moreira R. Targeting COPD: Advances on low-molecular-weight inhibitors of human neutrophil elastase. Medicinal Research Reviews. 2013;33(SUPPL. 1):E73-E101.

221. Lustig RH. Fructose: metabolic, hedonic, and societal parallels with ethanol. Journal of the American Dietetic Association. 2010;110(9):1307-21.

222. Lynch J, Durham W, Jangorbani M, Schuette S, Dillon E, Danesi C, et al. Spot determinations of urinary 3-methylhistidine isotope enrichment decay to identify age-related differences in protein degradation. Endocrine reviews. 2012;33(3).

223. Magnuson BA, Burdock GA, Doull J, Kroes RM, Marsh GM, Pariza MW, et al. Aspartame: A safety evaluation based on current use levels, regulations, and toxicological and epidemiological studies. Critical Reviews in Toxicology. 2007;37(8):629-727. doi: Doi 10.1080/10408440701516184. PubMed PMID: WOS:000249786200001.

224. Magrath G, Hartland BV, Govindji A, Kinmonth AL, Reckless J, Brenchley S, et al. Dietary recommendations for children and adolescent with diabetes: An implementation paper. Diabetic Medicine. 1993;10(9):874-85.

225. Malik VS, Hu FB. Sugar-sweetened beverages and health: Where does the evidence stand? American Journal of Clinical Nutrition. 2011;94(5):1161-2.

226. Mann J. Dietary advice for diabetics: a perspective from the United Kingdom. J Am Coll Nutr. 1986;5(1):1-7. Epub 1986/01/01. PubMed PMID: 3009586.

227. Manoguerra AS, Erdman AR, Booze LL, Christianson G, Wax PM, Scharman EJ, et al. Iron ingestion: An evidence-based consensus guideline for out-of-hospital management. Clinical Toxicology. 2005;43(6):553-70.

228. Mansour A, Hosseini S, Larijani B, Pajouhi M, Mohajeri-Tehrani MR. Nutrients related to GLP1 secretory responses. Nutrition. 2013;29(6):813-20.

229. Manuprakash SK, Varadarajshenoy K. Synthetic drinks and ill health in children. Indian Journal of Public Health Research and Development. 2012;3(2):116-9.

230. Martyn DM, McNulty BA, Nugent AP, Gibney MJ. Food additives and preschool children. Proceedings of the Nutrition Society. 2013;72(1):109-16.

231. Massougbodji J, Le Bodo Y, Fratu R, De Wals P. Reviews examining sugar-sweetened beverages and body weight: Correlates of their quality and conclusions. American Journal of Clinical Nutrition. 2014;99(5):1096-104.

232. Mattes RD, Shikany JM, Kaiser KA, Allison DB. Nutritively sweetened beverage consumption and body weight: A systematic review and meta-analysis of randomized experiments. Obesity Reviews. 2011;12(5):346-65.

233. McCarty MF. Optimizing exercise for fat loss. Medical hypotheses. 1995;44(5):325-30.

234. McKiernan F, Houchins JA, Mattes RD. Relationships between human thirst, hunger, drinking, and feeding. Physiology & behavior. 2008;94(5):700-8. doi: 10.1016/j.physbeh.2008.04.007. PubMed PMID: WOS:000257644000012.

235. Mehnert H. Sugar substitutes in the diabetic diet. Zuckeraustauschstoffe in der Diabetesdiaet. 1976;15:295-324.

236. Melanson KJ, Angelopoulos TJ, Nguyen V, Zukley L, Lowndes J, Rippe JM. High-fructose corn syrup, energy intake, and appetite regulation. American Journal of Clinical Nutrition. 2008;88(6):1738S-44S.

237. Meyer-Gerspach AC, Wölnerhanssen B, Beglinger C. Gut sweet taste receptors and their role in metabolism. Frontiers of Hormone Research2014. p. 123-33.

238. Miller P, Perez V. Low-calorie sweeteners and body weight and composition: A meta-analysis of randomized controlled trials and prospective cohorts. FASEB Journal. 2014;28(1).

239. Misra A, Sharma R, Gulati S, Joshi SR, Sharma V, Ghafoorunissa, et al. Consensus dietary guidelines for healthy living and prevention of obesity, the metabolic syndrome, diabetes, and related disorders in Asian Indians. Diabetes Technology and Therapeutics. 2011;13(6):683-94.

240. Moawad FJ, Veerappan GR, Wong RK. Eosinophilic esophagitis. Dig Dis Sci. 2009;54(9):1818-28. Epub 2009/06/26. doi: 10.1007/s10620-009-0873-6. PubMed PMID: 19554448.

241. Moeller SM, Fryhofer SA, Osbahr 3rd AJ, Robinowitz CB, Council on S, Public Health AMA. The effects of high fructose syrup. Journal of the American College of Nutrition. 2009;28(6):619-26.

242. Moon HJ, Jeya M, Kim IW, Lee JK. Biotechnological production of erythritol and its applications. Appl Microbiol Biotechnol. 2010;86(4):1017-25. Epub 2010/02/27. doi: 10.1007/s00253-010-2496-4. PubMed PMID: 20186409.

243. Moore MC. Drug evaluation: Tagatose in the treatment of type 2 diabetes and obesity. Current Opinion in Investigational Drugs. 2006;7(10):924-35.

244. Morgan RE. Does consumption of high-fructose corn syrup beverages cause obesity in children? Pediatric Obesity. 2013;8(4):249-54.

245. Mormann W. Silylation of cellulose with hexamethyldisilazane in ammonia - activation, catalysis, mechanism, properties. Cellulose. 2003;10(3):271-81. doi: 10.1023/a:1025100829371. PubMed PMID: WOS:000184690600008.

246. Morris DH, Stare FJ. Unproven diet therapies in the treatment of the chronic fatigue syndrome. Archives of family medicine. 1993;2(2):181-6.

247. Mortensen A. Sweeteners permitted in the European Union: Safety aspects. Scandinavian Journal of Food and Nutrition. 2006;50(3):104-16.

248. Moyer AE, Rodin J. FRUCTOSE AND BEHAVIOR - DOES FRUCTOSE INFLUENCE FOOD-INTAKE AND MACRONUTRIENT SELECTION. American Journal of Clinical Nutrition. 1993;58(5):S810-S4. PubMed PMID: WOS:A1993MF18500013.

249. Murray R, Frankowski B, Taras H. Are soft drinks a scapegoat for childhood obesity? The Journal of pediatrics. 2005;146(5):586-90. Epub 2005/05/05. doi: 10.1016/j.jpeds.2004.12.018. PubMed PMID: 15870659.

250. Murthy M. Nutraceuticals, functional foods and medical foods: Commentary and caveats. Journal of Nutraceuticals, Functional and Medical Foods. 1997;1(3):73-99.

251. Naniwadekar AS. Nutritional recommendations for patients with non-alcoholic fatty liver disease: An evidence based review. Practical Gastroenterology. 2010;34(2):8-16.

252. Nass J, Connolly S. Medullary carcinoma of the thyroid presenting as diarrhea in a patient meeting the Rome III criteria for irritable bowel syndrome. American Journal of Gastroenterology. 2011;106:S273.

253. Naumann K. Influence of chlorine substituents on biological activity of chemicals: A review. Pest Management Science. 2000;56(1):3-21.

254. Nestle M, Bal DG, Birt DF, Block G, Byers T, Foerster S, et al. Guidelines on diet, nutrition, and cancer prevention: Reducing the risk of cancer with healthy food choices and physical activity. Ca-A Cancer Journal for Clinicians. 1996;46(6):325-41.

255. Neuschwander-Tetri BA. Lifestyle Modification as the Primary Treatment of NASH. Clinics in Liver Disease. 2009;13(4):649-65.

256. Nielsen SJ, Popkin BM. Changes in beverage intake between 1977 and 2001. American Journal of Preventive Medicine. 2004;27(3):205-10.

257. Nomura K, Yamanouchi T. The role of fructose-enriched diets in mechanisms of nonalcoholic fatty liver disease. Journal of Nutritional Biochemistry. 2012;23(3):203-8.

258. Novak NL, Brownell KD. Taxation as prevention and as a treatment for obesity: The case of sugar-sweetened beverages. Current Pharmaceutical Design. 2011;17(12):1218-22.

259. Nseir W, Nassar F, Assy N. Soft drinks consumption and nonalcoholic fatty liver disease. World Journal of Gastroenterology. 2010;16(21):2579-88.

260. Nunn T, Williams J. Formulation of medicines for children. British Journal of Clinical Pharmacology. 2005;59(6):674-6.

261. O'Connor TM, Yang SJ, Nicklas TA. Beverage intake among preschool children and its effect on weight status. Pediatrics. 2006;118(4):E1010-E8. doi: 10.1542/peds.2005-2348. PubMed PMID: WOS:000240959300082.

262. Oh DK. Tagatose: properties, applications, and biotechnological processes. Appl Microbiol Biotechnol. 2007;76(1):1-8. Epub 2007/05/12. doi: 10.1007/s00253-007-0981-1. PubMed PMID: 17492284.

263. Olszewski PK, Levine AS. Central opioids and consumption of sweet tastants: when reward outweighs homeostasis. Physiology & behavior. 2007;91(5):506-12. Epub 2007/02/24. doi: 10.1016/j.physbeh.2007.01.011. PubMed PMID: 17316713.

264. Palmer AK, Ulbrich BC. The cult of culling. Fundamental and Applied Toxicology. 1997;38(1):7-22.

265. Pan A, Hu FB. Effects of carbohydrates on satiety: Differences between liquid and solid food. Current Opinion in Clinical Nutrition and Metabolic Care. 2011;14(4):385-90.

266. Panpatil VV, Polasa K. Assessment of stevia (Stevia rebaudiana)-natural sweetener: A review. Journal of Food Science and Technology. 2008;45(6):467-73.

267. Parmar J, Gasevic D, Corber E, Punthankee Z, Lear S. Investigating lifestyle factors associated with obesity in South Asian children. Obesity Reviews. 2014;15:245.

268. Pasha SF, Sharma VK, Crowell MD. Current concepts and treatment options in eosinophilic esophagitis. Current Opinion in Investigational Drugs. 2006;7(11):992-6.

269. Pasinetti GM, Eberstein JA. Metabolic syndrome and the role of dietary lifestyles in Alzheimer's disease. Journal of Neurochemistry. 2008;106(4):1503-14.

270. Passos RM, Se AB, Wolff VL, Nobrega YKM, Hermes-Lima M. Pizza and pasta help students learn metabolism. American Journal of Physiology - Advances in Physiology Education. 2006;30(2):89-93.

271. Patel Y, Shukla A, Saini V, Shrimal N, Sharma P. Chewing gum as a drug delivery system. International Journal of Pharmaceutical Sciences and Research. 2011;2(4):748-57.

272. Pater C. Hyperuricemia and hypertension - a causal relationship ignored for all too long. Current Hypertension Reviews. 2011;7(1):41-53.

273. Patil BS, Tate SS, Kulkarni U, Hariprasanna RC, Wadageri GV. Development and in-vitro evaluation of mucoadhesive buccal tablets of Tizanidine hydrochloride using natural polymer xanthan gum. International Journal of Pharmaceutical Sciences Review and Research. 2011;8(2):140-6.

274. Patra F, Tomar SK, Arora S. Technological and functional applications of low-calorie sweeteners from lactic acid bacteria. J Food Sci. 2009;74(1):R16-23. Epub 2009/02/10. doi: 10.1111/j.1750-3841.2008.01005.x. PubMed PMID: 19200114.

275. Payne AN, Chassard C, Lacroix C. Gut microbial adaptation to dietary consumption of fructose, artificial sweeteners and sugar alcohols: Implications for host-microbe interactions contributing to obesity. Obesity Reviews. 2012;13(9):799-809.

276. Penson RT. Sugar fuels cancer. Cancer. 2009;115(5):918-21. Epub 2009/01/22. doi: 10.1002/cncr.24125. PubMed PMID: 19156846.

277. Pereira L. A review of the nutrient composition of selected edible seaweeds. Seaweed: Ecology, Nutrient Composition and Medicinal Uses2011. p. 15-49.

278. Perez-Morales E, Bacardi-Gascon M, Jimenez-Cruz A. Sugar-sweetened beverage intake before 6 years of age and weight or BMI status among older children; systematic review of prospective studies. Nutr Hosp. 2013;28(1):47-51. Epub 2013/07/03. doi: 10.3305/nh.2013.28.1.6247. PubMed PMID: 23808429.

279. Perito ER, Rodriguez LA, Lustig RH. Dietary treatment of nonalcoholic steatohepatitis. Current Opinion in Gastroenterology. 2013;29(2):170-6.

280. Pillai O, Nair V, Poduri R, Panchagnula R. Transdermal iontophoresis. Part II: Peptide and protein delivery. Methods and Findings in Experimental and Clinical Pharmacology. 1999;21(3):229-40.

281. Pomeranz JL. Advanced policy options to regulate sugar-sweetened beverages to support public health. Journal of Public Health Policy. 2012;33(1):75-88.

282. Pomeranz JL, Munsell CR, Harris JL. Energy drinks: an emerging public health hazard for youth. J Public Health Policy. 2013;34(2):254-71. Epub 2013/03/15. doi: 10.1057/jphp.2013.6. PubMed PMID: 23486464.

283. Popkin BM. Synthesis and implications: China's nutrition transition in the context of changes across other low- and middle-income countries. Obesity Reviews. 2014;15(S1):60-7.

284. Porrata Maury C, Sanchez Cruz J, Correa Corrales V, Abuin Landin A, Hernandez-Triana M, Dacosta-Calheiros RV, et al. Ma-Pi 2 macrobiotic diet intervention in adults with type 2 diabetes mellitus. MEDICC Review. 2009;11(4):29-35.

285. Potvin Kent M, Wanless A. The influence of the Children's Food and Beverage Advertising Initiative: Change in children's exposure to food advertising on television in Canada between 2006-2009. International Journal of Obesity. 2014;38(4):558-62.

286. Pratik S, Asif K, Ramana MV, Mitul P, Mahesh K. Chewing gum: A modern era of drug delivery. International Research Journal of Pharmacy. 2011;2(10):7-12.

287. Raben A, Moller BK, Vasilaras TH, Moller AC, Flint A, Astrup A. Sucrose vs artificial sweeteners: Differences in 24 hour substrate oxidation rates, but not in energy expenditure after 10 weeks ad libitum intake in overweight subjects. Obesity Reviews. 2011;12:79.

288. Rani AS, Satyakala M, Murty US. Stevia rebaudiana, bio-sweetener of the future: A review. Asian Journal of Microbiology, Biotechnology and Environmental Sciences. 2004;6(3):453-60.

289. Reddy CS, Sowmya C, Keerthi K, Priya NV, Sandhya R. An overview of fast dissolving tablets. International Journal of Pharmacy and Technology. 2012;4(2):2119-34.

290. Reddy V, Ghaffari G. Eosinophilic esophagitis: review of nonsurgical treatment modalities. Allergy and asthma proceedings : the official journal of regional and state allergy societies. 2013;34(5):421-6. Epub 2013/09/04. doi: 10.2500/aap.2013.34.3690. PubMed PMID: 23998238.

291. Reed DR, Bachmanov AA, Beauchamp GK, Tordoff MG, Price RA. Heritable variation in food preferences and their contribution to obesity. Behavior Genetics. 1997;27(4):373-87. doi: Doi 10.1023/A:1025692031673. PubMed PMID: WOS:A1997XX89200009.

292. Rees CJ. Lifestyle modifications for reflux. Effects, Diagnosis and Management of Extra-Esophageal Reflux2012. p. 123-30.

293. Reiner Z, Catapano AL, De Backer G, Graham I, Taskinen MR, Wiklund O, et al. ESC/EAS Guidelines for the management of dyslipidaemias. European Heart Journal. 2011;32(14):1769-818.

294. Renwick AG, Molinary SV. Sweet-taste receptors, low-energy sweeteners, glucose absorption and insulin release. British Journal of Nutrition. 2010;104(10):1415-20.

295. Richelsen B. Sugar-sweetened beverages and cardio-metabolic disease risks. Current Opinion in Clinical Nutrition and Metabolic Care. 2013;16(4):478-84.

296. Righetti PG. Capillary electrophoretic analysis of proteins and peptides of biomedical and pharmacological interest. Biopharmaceutics & drug disposition. 2001;22(7-8):337-51. Epub 2002/02/09. PubMed PMID: 11835253.

297. Rippe JM. The metabolic and endocrine response and health implications of consuming sugar-sweetened beverages: findings from recent randomized controlled trials. Advances in nutrition (Bethesda, Md). 2013;4(6):677-86.

298. Rippe JM, Angelopoulos TJ. Sucrose, high-fructose corn syrup, and fructose, their metabolism and potential health effects: What do we really know? Advances in Nutrition. 2013;4(2):236-45.

299. Rizkalla SW. Health implications of fructose consumption: A review of recent data. Nutrition and Metabolism. 2010;7.

300. Roehrig KL. THE INFLUENCE OF FOOD ON FOOD-INTAKE - METHODOLOGICAL PROBLEMS AND MECHANISMS OF ACTION. Critical Reviews in Food Science and Nutrition. 1991;30(6):575-97. PubMed PMID: WOS:A1991GM91500002.

301. Roehrig KL. The influence of food on food intake: methodological problems and mechanisms of action. Crit Rev Food Sci Nutr. 1991;30(6):575-97. Epub 1991/01/01. doi: 10.1080/10408399109527557. PubMed PMID: 1741952.

302. Rolls BJ. Carbohydrates, fats, and satiety. The American journal of clinical nutrition. 1995;61(4 Suppl):960s-7s. Epub 1995/04/01. PubMed PMID: 7900695.

303. Rosado EL, Monteiro JBR. Obesity and diet macronutrients substitution. Revista de Nutricao. 2001;14(2):145-52.

304. Rulis AM, Levitt JA. FDA'S food ingredient approval process. Safety assurance based on scientific assessment. Regulatory Toxicology and Pharmacology. 2009;53(1):20-31.

305. Russell W, Baka A, Bjorck I, Delzenne N, Gao D, Griffiths H, et al. Nutritional management of blood glucose levels. Annals of Nutrition and Metabolism. 2013;63:1339-40.

306. Russell WR, Baka A, Bjorck I, Delzenne N, Gao D, Griffiths HR, et al. Nutritional management of postprandial glycaemia as a diabetes prevention and management strategy. Journal of Diabetes. 2013;5:82.

307. Russell WR, Baka A, Bjorck I, Delzenne N, Gao D, Griffiths HR, et al. Impact of diet composition on blood glucose regulation. Crit Rev Food Sci Nutr. 2013. Epub 2013/11/14. doi: 10.1080/10408398.2013.792772. PubMed PMID: 24219323.

308. Rutledge AC, Adeli K. Fructose and the Metabolic Syndrome: Pathophysiology and Molecular Mechanisms. Nutrition Reviews. 2007;65(SUPPL.1):S13-S23.

309. Saarni U, Saarni H. Xylitol for messrooms--a method worth trying to prevent caries among seafarers. Bulletin of the Institute of Maritime and Tropical Medicine in Gdynia. 1997;48(1-4):91-7.

310. Salame E, Mallat S, Kassab R, Berbari A. Diet and essential hypertension. Journal Medical Libanais. 2005;53(4):220-8.

311. Salamé E, Mallat S, Kassab R, Berbari A. Diet and essential hypertension. Diète et hypertension artérielle essentielle. 2005;53(4):220-8.

312. Salunkhe VR, Bhise SB. Stevia rebaudiana: An alternative to synthetic sweeteners. Indian Drugs. 2010;47(2):5-13.

313. Sandeep V. Type 2 diabetes. Annals of Internal Medicine. 2010;152(5):ITC31-ITC316.

314. Sandrou DK, Arvanitoyannis IS. Low-fat/calorie foods: Current state and perspectives. Critical Reviews in Food Science and Nutrition. 2000;40(5):427-47.

315. Schiffman SS, Rother KI. Sucralose, a synthetic organochlorine sweetener: Overview of biological issues. Journal of Toxicology and Environmental Health - Part B: Critical Reviews. 2013;16(7):399-451.

316. Schmandke H. Sweet-tasting steviol glycoside derivatives with antihyperglycaemic and antihypertensive effects. Ernahrungs Umschau. 2004;51(11):455-8+38.

317. Schneider LM, Schermbeck RM, Chriqui JF, Chaloupka FJ. The Extent to Which School District Competitive Food and Beverage Policies Align with the 2010 Dietary Guidelines for Americans: Implications for Federal Regulations. Journal of the Academy of Nutrition and Dietetics. 2012;112(6):892-6.

318. Seidemann J. Stevioside, an interesting natural sweetening agent. Die Nahrung. 1976;20(6):675-9.

319. Semchyshyn HM. Fructation in vivo: Detrimental and protective effects of fructose. BioMed Research International. 2013;2013.

320. Senard JM, Montastruc JL. Recent advances in the pharmacology of antimigraine drugs. Drugs of Today. 1992;28(2):131-43.

321. Serio L. Stevia rebaudiana, an alternative to sugar. Phytotherapie. 2010;8(1):26-32.

322. Serrano JC, Sanchez Gonzalez I. Trends in functional foods against obesity: Functional ingredients, technologically modified foods and complete diets. Revista Espanola de Nutricion Comunitaria. 2008;14(3):193-200.

323. Servant G, Tachdjian C, Li X, Karanewsky DS. The sweet taste of true synergy: Positive allosteric modulation of the human sweet taste receptor. Trends in Pharmacological Sciences. 2011;32(11):631-6.

324. Shah KR, Mehta TA. Medicated chewing gum-a mobile oral drug delivery system. International Journal of PharmTech Research. 2014;6(1):35-48.

325. Sharkey JR, Johnson CM, Dean WR. Nativity is associated with sugar-sweetened beverage and fast-food meal consumption among Mexican-origin women in Texas border colonias. Nutrition Journal. 2011;10(1).

326. Shet N, Vaidya I. Taste masking: A pathfinder for bitter drugs. International Journal of Pharmaceutical Sciences Review and Research. 2013;18(2):1-12.

327. Shwide-Slavin C, Anekwe AV. Sugar, sugar everywhere Integrating the current evidence on nonnutritive sweeteners. Agro Food Industry Hi-Tech. 2013;24(3):20-3. PubMed PMID: WOS:000324215400005.

328. Sievenpiper JL. Fructose: Where does the truth lie? Journal of the American College of Nutrition. 2012;31(3):149-51.

329. Sievenpiper JL, De Souza RJ. Are sugar-sweetened beverages the whole story? American Journal of Clinical Nutrition. 2013;98(2):261-3.

330. Sievenpiper JL, de Souza RJ, Mirrahimi A, Yu ME, Carleton AJ, Beyene J, et al. Effect of fructose on body weight in controlled feeding trials: a systematic review and meta-analysis. Ann Intern Med. 2012;156(4):291-304. Epub 2012/02/22. doi: 10.7326/0003-4819-156-4-201202210-00007. PubMed PMID: 22351714.

331. Sigoillot M, Brockhoff A, Meyerhof W, Briand L. Sweet-taste-suppressing compounds: current knowledge and perspectives of application. Appl Microbiol Biotechnol. 2012;96(3):619-30. Epub 2012/09/18. doi: 10.1007/s00253-012-4387-3. PubMed PMID: 22983596.

332. Silverman AG. Overnutrition in the diabetic patient. Mount Sinai Journal of Medicine. 1987;54(3):211-6.

333. Silvis N. Nutritional recommendations for individuals with diabetes mellitus. South African Medical Journal. 1992;81(3):162-6.

334. Simmons AL, Schlezinger JJ, Corkey BE. What Are We Putting in Our Food That Is Making Us Fat? Food Additives, Contaminants, and Other Putative Contributors to Obesity. Current Obesity Reports. 2014;3(2):273-85.

335. Simon PA, Lightstone AS, Baldwin S, Kuo T, Shih M, Fielding JE. Declines in sugar-sweetened beverage consumption among children in los angeles county, 2007 and 2011. Preventing Chronic Disease. 2013;10(8).

336. Simopoulos AP. Dietary omega-3 fatty acid deficiency and high fructose intake in the development of metabolic syndrome, brain metabolic abnormalities, and non-alcoholic fatty liver disease. Nutrients. 2013;5(8):2901-23. Epub 2013/07/31. doi: 10.3390/nu5082901. PubMed PMID: 23896654; PubMed Central PMCID: PMCPmc3775234.

337. Slavin J. Beverages and body weight: Challenges in the evidence-based review process of the Carbohydrate Subcommittee from the 2010 Dietary Guidelines Advisory Committee. Nutrition Reviews. 2012;70(SUPPL/2):S111-S20.

338. Sleator RD, Hill C. Molecular analysis of the microbial food safety implications of food reformulations for improved health. Foodborne Pathog Dis. 2008;5(4):499-504. Epub 2008/08/01. doi: 10.1089/fpd.2008.0089. PubMed PMID: 18666862.

339. Sloboda DM, Li M, Patel R, Clayton ZE, Yap C, Vickers MH. Early life exposure to fructose and offspring phenotype: Implications for long term metabolic homeostasis. Journal of Obesity. 2014;2014.

340. Small DM. Individual differences in the neurophysiology of reward and the obesity epidemic. International Journal of Obesity. 2009;33:S44-S8. doi: 10.1038/ijo.2009.71. PubMed PMID: WOS:000267566100009.

341. Smeets PAM, Erkner A, de Graaf C. Cephalic phase responses and appetite. Nutrition Reviews. 2010;68(11):643-55. doi: 10.1111/j.1753-4887.2010.00334.x. PubMed PMID: WOS:000283323300001.

342. Smirnova MG. Physiologic and toxis effects on organism sweetening stevioside (review). Voprosy Pitaniia. 2001;70(4):41-4.

343. Smirnova MG. [Study of physiological and toxic effects of a sweetening agent stevioside (review of the literature)]. Vopr Pitan. 2001;70(4):41-4. Epub 2001/09/12. PubMed PMID: 11550460.

344. Soffritti M, Padovani M, Tibaldi E, Falcioni L, Manservisi F, Belpoggi F. Effects of Aspartame: The Urgent Need for Regulatory Re- Evaluation. American journal of industrial medicine. 2014;57(4):383-97. doi: 10.1002/ajim.22296. PubMed PMID: WOS:000332217900001.

345. Solnick SJ, Hemenway D. The 'twinkie defense': The relationship between carbonated non-diet soft drinks and violence perpetration among boston high school students. Injury Prevention. 2012;18(4):259-63.

346. Sorensen LB, Moller P, Flint A, Martens M, Raben A. Effect of sensory perception of foods on appetite and food intake: a review of studies on humans. International journal of obesity and related metabolic disorders : journal of the International Association for the Study of Obesity. 2003;27(10):1152-66. Epub 2003/09/27. doi: 10.1038/sj.ijo.0802391. PubMed PMID: 14513063.

347. Souberbielle JC, Guinot M, Fink E, Cristol R, Perin L, Le Bouc Y. Growth hormone and doping. Immuno-Analyse et Biologie Specialisee. 2001;16(3):183-8.

348. Spratt Jr JS. Your behavior and cancer. Missouri Medicine. 1974;71(1):22A-4.

349. Sprous D, Palmer KR. The T1R2/T1R3 sweet receptor and TRPM5 ion channel taste targets with therapeutic potential. Progress in molecular biology and translational science. 2010;91:151-208. Epub 2010/08/10. doi: 10.1016/s1877-1173(10)91006-5. PubMed PMID: 20691962.

350. Sreekanth J, Lakshmi PK. Formulation development of fast releasing oral thin films of levocetrizine dihydrochloride with eudragit EPO and optimisation through taguchi orthogonal experimental design. International Journal of Pharmaceutical Sciences Review and Research. 2011;11(1):115-23.

351. Stanhope KL, Havel PJ. Endocrine and metabolic effects of consuming beverages sweetened with fructose, glucose, sucrose, or high-fructose corn syrup. American Journal of Clinical Nutrition. 2008;88(6):1733S-7S.

352. Stanhope KL, Havel PJ. Fructose consumption: Potential mechanisms for its effects to increase visceral adiposity and induce dyslipidemia and insulin resistance. Current Opinion in Lipidology. 2008;19(1):16-24.

353. Stanhope KL, Schwarz JM, Havel PJ. Adverse metabolic effects of dietary fructose: results from the recent epidemiological, clinical, and mechanistic studies. Curr Opin Lipidol. 2013;24(3):198-206. Epub 2013/04/19. doi: 10.1097/MOL.0b013e3283613bca. PubMed PMID: 23594708.

354. Sternini C. In Search of a Role for Carbonation: Is This a Good or Bad Taste? Gastroenterology. 2013;145(3):500-3. doi: Doi 10.1053/J.Gastro.2013.07.018. PubMed PMID: WOS:000323613700009.

355. Stevens HC. Fructose impacts on gut microbiota and obesity by A. N. Payne etal. Obesity Reviews. 2012;13(12):1182-3.

356. Steyn NP, Temple NJ. The Dilemma of Sugar-Sweetened Beverages. Current Nutrition Reports. 2013;2(3):127-8.

357. Stolle A, Sperner B, Krausse HG. Harmonisation of selected food-related regulations. Archiv Fur Lebensmittelhygiene. 1997;48(5):99-104. PubMed PMID: WOS:A1997YJ47500001.

358. Stookey JD. Drinking water and weight management. Nutrition Today. 2010;45(6 SUPPL.):S7-S12.

359. Strata A, Magnati G. Nutritional principles for the pregnant diabetic. Minerva Endocrinologica. 1994;19(2):73-7.

360. Struck S, Jaros D, Brennan CS, Rohm H. Sugar replacement in sweetened bakery goods. International Journal of Food Science and Technology. 2014.

361. Stubbs J, Ferres S, Horgan G. Energy density of foods: Effects on energy intake. Critical Reviews in Food Science and Nutrition. 2000;40(6):481-515.

362. Stubbs RJ, Prentice AM, James WP. Carbohydrates and energy balance. Annals of the New York Academy of Sciences. 1997;819:44-69. Epub 1997/05/23. PubMed PMID: 9186760.

363. Supuran CT. Therapeutic applications of the carbonic anhydrase inhibitors. Therapy. 2007;4(3):355-78.

364. Supuran CT. Carbonic anhydrases: Novel therapeutic applications for inhibitors and activators. Nature Reviews Drug Discovery. 2008;7(2):168-81.

365. Swartz JJ, Braxton D, Viera AJ. Calorie menu labeling on quick-service restaurant menus: An updated systematic review of the literature. International Journal of Behavioral Nutrition and Physical Activity. 2011;8.

366. Swithers SE, Martin AA, Davidson TL. High-intensity sweeteners and energy balance. Physiology & behavior. 2010;100(1):55-62. Epub 2010/01/12. doi: 10.1016/j.physbeh.2009.12.021. PubMed PMID: 20060008; PubMed Central PMCID: PMCPmc2855968.

367. Talbot JM, Fisher KD. The need for special foods and sugar substitutes by individuals with diabetes mellitus. Diabetes Care. 1978;1(4):231-40. Epub 1978/07/01. PubMed PMID: 400133.

368. Tappy L, Egli L, Lecoultre V, Schneider P. Effects of fructose-containing caloric sweeteners on resting energy expenditure and energy efficiency: A review of human trials. Nutrition and Metabolism. 2013;10(1).

369. Tappy L, Lê KA, Tran C, Paquot N. Fructose and metabolic diseases: New findings, new questions. Nutrition. 2010;26(11-12):1044-9.

370. Tappy L, Mittendorfer B. Fructose toxicity: Is the science ready for public health actions? Current Opinion in Clinical Nutrition and Metabolic Care. 2012;15(4):357-61.

371. Teixeira TFS, Collado MC, Ferreira CLLF, Bressan J, Peluzio MCG. Potential mechanisms for the emerging link between obesity and increased intestinal permeability. Nutrition Research. 2012;32(9):637-47.

372. Temussi P. The history of sweet taste: Not exactly a piece of cake. Journal of Molecular Recognition. 2006;19(3):188-99.

373. Temussi P. The Sweet Taste Receptor: A Single Receptor with Multiple Sites and Modes of Interaction. 2007. p. 199-239.

374. Temussi PA. Natural sweet macromolecules: How sweet proteins work. Cellular and Molecular Life Sciences. 2006;63(16):1876-88.

375. Temussi PA. New insights into the characteristics of sweet and bitter taste receptors. International review of cell and molecular biology. 2011;291:191-226. Epub 2011/10/25. doi: 10.1016/b978-0-12-386035-4.00006-9. PubMed PMID: 22017977.

376. Terry-Mcelrath YM, O'Malley PM, Johnston LD. Factors affecting sugar-sweetened beverage availability in competitive venues of us secondary schools. Journal of School Health. 2012;82(1):44-55.

377. Thorat YS, Gonjari ID, Hosmani AH. Solubility enhancement techniques: A review on conventional and novel approaches. International Journal of Pharmaceutical Sciences and Research. 2011;2(10):2501-13.

378. Thornley S, McRobbie H, Jackson G. The New Zealand sugar (fructose) fountain: Time to turn the tide? New Zealand Medical Journal. 2010;123(1311):58-64.

379. Toeller M, Lion S. Survey of the management of diet in 26 diabetes centres in Europe. Diabetic medicine : a journal of the British Diabetic Association. 1987;4(2):129-34. Epub 1987/03/01. PubMed PMID: 2952429.

380. Torloni MR, Nakamura MU, Megale A, Sanchez VHS, Mano C, Fusaro AS, et al. The use of sweeteners in pregnancy: An analysis of products available in Brazil. O uso de adoçantes na gravidez: Uma análise dos produtos disponíveis no Brasil. 2007;29(5):267-75.

381. Trang J, Tran K, Lam R, Wu G. Metabolic syndrome awareness in Asian Americans and internet survey. Endocrine reviews. 2011;32(3).

382. Treesukosol Y, Smith KR, Spector AC. The functional role of the T1R family of receptors in sweet taste and feeding. Physiology and Behavior. 2011;105(1):14-26.

383. Trepanowski JF, Bloomer RJ. The impact of religious fasting on human health. Nutrition Journal. 2010;9(1).

384. Tyagi S, Mishra NC, Ray AK, editors. Bio-refinery based on Indian paper industry wastes2011.

385. Uplinger N. The controversy continues: Nutritional management of the pregnancy complicated by diabetes. Current Diabetes Reports. 2009;9(4):291-5.

386. Vaughan L. Dietary guidelines for the management of diabetes. Nursing standard (Royal College of Nursing (Great Britain) : 1987). 2005;19(44):56-64; quiz 6.

387. Ventura AK, Mennella JA. Innate and learned preferences for sweet taste during childhood. Current Opinion in Clinical Nutrition and Metabolic Care. 2011;14(4):379-84.

388. Verma AK, Sachan AK. A review on fast dissolving tablet as an efficient technique for oral drug delivery. Journal of Chemical and Pharmaceutical Sciences. 2013;6(1):29-34.

389. Vos MB, Welsh J. Childhood obesity: Update on predisposing factors and prevention strategies. Current Gastroenterology Reports. 2010;12(4):280-7.

390. Wallinga D, Schoonover H, Muller M. Considering the contribution of US agricultural policy to the obesity epidemic: Overview and opportunities. Journal of Hunger and Environmental Nutrition. 2009;4(1):3-19.

391. Walpole J, Pittinger T, Hutsler D, Sadie C, Reed M. A descriptive study to determine if severely burned patients achieve their target caloric and protein goals within five days of injury. Journal of Burn Care and Research. 2014;35:S179.

392. Watanabe H, Ohe H, Nakagawa S, Takada H, Ebisui K, Hirayama T, et al. [A workshop on the high risk group and the preventive oncology of renal cell carcinoma]. Hinyokika kiyo Acta urologica Japonica. 1992;38(2):237-53. Epub 1992/02/01. PubMed PMID: 1561964.

393. Weed DL, Althuis MD, Mink PJ. Quality of reviews on sugar-sweetened beverages and health outcomes: A systematic review. American Journal of Clinical Nutrition. 2011;94(5):1340-7.

394. Wellendorph P, Johansen LD, Brauner-Osborne H. Molecular pharmacology of promiscuous seven transmembrane receptors sensing organic nutrients. Molecular Pharmacology. 2009;76(3):453-65.

395. Welsh JA, Cunningham SA. The role of added sugars in pediatric obesity. Pediatric Clinics of North America. 2011;58(6):1455-66.

396. Welsh JA, Lundeen EA, Stein AD. The sugar-sweetened beverage wars: Public health and the role of the beverage industry. Current Opinion in Endocrinology, Diabetes and Obesity. 2013;20(5):401-6.

397. West KM. Prevention and therapy of diabetes mellitus. Nutrition Reviews. 1975;33(7):193-8.

398. Westin JB. Ingestion of carcinogenic N-nitrosamines by infants and children. Archives of environmental health. 1990;45(6):359-63. Epub 1990/11/01. doi: 10.1080/00039896.1990.10118755. PubMed PMID: 2270955.

399. Wharton CM, Hampl JS. Beverage consumption and risk of obesity among Native Americans in Arizona. Nutr Rev. 2004;62(4):153-9. Epub 2004/05/15. PubMed PMID: 15141431.

400. Whatley Blum JE, Davee AM, Beaudoin CM, Jenkins PL, Kaley LA, Wigand DA. Reduced Availability of Sugar-sweetened Beverages and Diet Soda Has a Limited Impact on Beverage Consumption Patterns in Maine High School Youth. Journal of Nutrition Education and Behavior. 2008;40(6):341-7.

401. Wheless JW. Managing severe epilepsy syndromes of early childhood. J Child Neurol. 2009;24(8 Suppl):24S-32S; quiz 3S-6S. Epub 2009/08/15. doi: 10.1177/0883073809338153. PubMed PMID: 19666880.

402. White JS. Straight talk about high-fructose corn syrup: What it is and what it ain't. American Journal of Clinical Nutrition. 2008;88(6):1716S-21S.

403. White JS. Misconceptions about high-fructose corn syrup: Is it uniquely responsible for obesity, reactive dicarbonyl compounds, and advanced glycation endproducts? Journal of Nutrition. 2009;139(6):1219S-27S.

404. Whitehouse CR, Boullata J, McCauley LA. The potential toxicity of artificial sweeteners. AAOHN J. 2008;56(6):251-9; quiz 60-1. Epub 2008/07/09. PubMed PMID: 18604921.

405. Widen E, Siega-Riz AM. Prenatal nutrition: A practical guide for assessment and counseling. Journal of Midwifery and Women's Health. 2010;55(6):540-9.

406. Widowski TM, Torrey S, Bench CJ, Gonyou HW. Development of ingestive behaviour and the relationship to belly nosing in early-weaned piglets. Applied Animal Behaviour Science. 2008;110(1-2):109-27. doi: 10.1016/j.applanim.2007.04.010. PubMed PMID: WOS:000253658300008.

407. Wiernsperger N, Geloen A, Rapin JR. Fructose and cardiometabolic disorders: The controversy will, and must, continue. Clinics. 2010;65(7):729-38.

408. Wojcicki JM. Healthy hospital food initiatives in the United States: Time to ban sugar sweetened beverages to reduce childhood obesity. Acta Paediatrica, International Journal of Paediatrics. 2013;102(6):560-1.

409. Woodward-Lopez G, Kao J, Ritchie L. To what extent have sweetened beverages contributed to the obesity epidemic? Public Health Nutrition. 2011;14(3):499-509.

410. Wylie-Rosett J, Segal-Isaacson CJ, Segal-Isaacson A. Carbohydrates and increases in obesity: does the type of carbohydrate make a difference? Obesity research. 2004;12 Suppl 2:124S-9S.

411. Wystrychowski G, Zukowska-Szczechowska E, Obuchowicz E, Grzeszczak W, Wystrychowski A. [Carbohydrate sweeteners and obesity]. Przegla̧d lekarski. 2012;69(4):157-62.

412. Yamada M, Murakami K, Sasaki S, Takahashi Y, Okubo H. Soft Drink Intake Is Associated with Diet Quality Even among Young Japanese Women with Low Soft Drink Intake. Journal of the American Dietetic Association. 2008;108(12):1997-2004.

413. Ye J, Cui X, Loraine A, Bynum K, Kim NC, White G, et al. Methods for nutrigenomics and longevity studies in Drosophila effects of diets high in sucrose, palmitic acid, soy, or beef. Methods in Molecular Biology2007. p. 111-41.

414. Yengopal V, Mickenautsch S. Caries preventive effect of casein phosphopeptide-amorphous calcium phosphate (CPP-ACP): a meta-analysis. Acta Odontologica Scandinavica. 2009;67(6):321-32. doi: 10.1080/00016350903160563. PubMed PMID: WOS:000272142400001.

415. Zhang YH, An T, Zhang RC, Zhou Q, Huang Y, Zhang J. Very high fructose intake increases serum LDL-cholesterol and total cholesterol: A meta-analysis of controlled feeding trials. Journal of Nutrition. 2013;143(9):1391-8.

416. Zheng M, Allman-Farinelli M, Marks G, Toelle B, Cowell C, Heitmann B, et al. Intake of liquid versus solid energy and change in weight status in Australian children. Obesity Reviews. 2014;15:234-5.

417. Zygler A, Wasik A, Namieśnik J. Analytical methodologies for determination of artificial sweeteners in foodstuffs. TrAC - Trends in Analytical Chemistry. 2009;28(9):1082-102.

418. Position of the American Dietetic Association: Fat replacers. Journal of the American Dietetic Association. 2005;105(2):266-75.

419. Carbajal R. Nonpharmacologic management of pain in neonates. Traitement non pharmacologique de la douleur du nouveau-né. 2005;12(1):110-6.

420. Ferenczi EA, Asaria P, Hughes AD, Chaturvedi N, Francis DP. Can a statin neutralize the cardiovascular risk of unhealthy dietary choices? American Journal of Cardiology. 2010;106(4):587-92.

421. Flaisher-Grinberg S, Einat H. Mice models for the manic pole of bipolar disorder. 2009. p. 297-326.

422. Hoebel BG, Avena NM, Bocarsly ME, Rada P. Natural addiction: a behavioral and circuit model based on sugar addiction in rats. J Addict Med. 2009;3(1):33-41. Epub 2009/03/01. doi: 10.1097/ADM.0b013e31819aa621. PubMed PMID: 21768998.

423. Kruger CL, Whittaker MH, Frankos VH, Trimmer GW. 90-Day oral toxicity study of D-tagatose in rats. Regulatory Toxicology and Pharmacology. 1999;29(2 I):S1-S10.

424. Malkesman O, Weller A. Two different putative genetic animal models of childhood depression-A review. Progress in Neurobiology. 2009;88(3):153-69.

425. Oser BL. The rat as a model for human toxicological evaluation. Journal of Toxicology and Environmental Health. 1981;8(4):521-42.

426. Rolls BJ, Miller DL. Is the low-fat message giving people a license to eat more? Journal of the American College of Nutrition. 1997;16(6):535-43.

427. Sudesh K, Bhubalan K, Chuah JA, Kek YK, Kamilah H, Sridewi N, et al. Synthesis of polyhydroxyalkanoate from palm oil and some new applications. Applied Microbiology and Biotechnology. 2011;89(5):1373-86. doi: Doi 10.1007/S00253-011-3098-5. PubMed PMID: WOS:000287199900011.

**FULL TEXT ARTICLES EXCLUDED (n=35)**

*Health effects other than weight outcomes (n=29)*

428. Sweetening agents. Pediatrics and Related Topics. 1969;8(1):95-7.

429. Position of the american dietetic association: Use of nutritive and nonnutritive sweeteners. Journal of the American Dietetic Association. 2004;104(2):255-75.

430. AlDeeb OAA, Mahgoub H, Foda NH. Sucralose. 2013. p. 423-62.

431. Arlin M. Controversies in nutrition. A brief review. Nursing Clinics of North America. 1979;14(2):199-214.

432. Aune D. Soft drinks, aspartame, and the risk of cancer and cardiovascular disease. The American journal of clinical nutrition. 2012;96(6):1249-51. Epub 2012/11/09. doi: 10.3945/ajcn.112.051417. PubMed PMID: 23134894; PubMed Central PMCID: PMC3497921.

433. Blundell JE, Green SM. Effect of sucrose and sweeteners on appetite and energy intake. International journal of obesity and related metabolic disorders : journal of the International Association for the Study of Obesity. 1996;20 Suppl 2:S12-7. Epub 1996/03/01. PubMed PMID: 8646266.

434. Booth DA. Mechanisms from Models Actual Effects from Real Life - the Zero-Calorie Drink-Break Option. Appetite. 1988;11(SUPPL. 1):94-102. doi: Doi 10.1016/S0195-6663(88)80053-9. PubMed PMID: WOS:A1988Q027700016.

435. Bukhamseen F, Novotny L. Artificial sweeteners and sugar substitutes -some properties and potential health benefits and risks. Research Journal of Pharmaceutical, Biological and Chemical Sciences. 2014;5(1):638-49.

436. Crapo PA. Use of alternative sweeteners in diabetic diet. Diabetes Care. 1988;11(2):174-82. Epub 1988/02/01. PubMed PMID: 3289865.

437. Durán Agüero S, Cordón A K, Rodríguez N MP. Non-nutritive sweeteners risks, appetite and weight gain. Revista Chilena de Nutricion. 2013;40(3):309-14.

438. Franz MJ, Powers MA, Leontos C, Holzmeister LA, Kulkarni K, Monk A, et al. The evidence for medical nutrition therapy for type 1 and type 2 diabetes in adults. Journal of the American Dietetic Association. 2010;110(12):1852-89.

439. García-Almeida JM, Fdez GMC, Alemán JG. A current and global review of sweeteners. Regulatory aspects. Una visión global y actual de los edulcorantes Aspectos de regulación. 2013;28(SUPPL.4):17-31.

440. Gaßmann B. Sweeteners and metabolic syndrome. Süßungsmittel und metabolisches syndrom. 2005;52(12):476-81+4.

441. Greenwood DC, Threapleton DE, Evans CEL, Cleghorn CL, Nykjaer C, Woodhead C, et al. Association between sugar-sweetened and artificially sweetened soft drinks and type 2 diabetes: systematic review and dose-response meta-analysis of prospective studies. British Journal of Nutrition. 2014.

442. Gupta S, Mahajan V, Mahajan S, Tandon VR. Artificial sweeteners. JK Science. 2011;14(1):2-4.

443. Ha MA, Ha TK, Lean ME. Role of intense sweeteners in diabetes management. World review of nutrition and dietetics. 1999;85:88-97.

444. Henin N. Intake of sweeteners, physiological and nutritional aspects. International Sugar Journal. 2001;103(1232):346-51. PubMed PMID: WOS:000170462700026.

445. Magnuson B. Aspartame-facts and fiction. New Zealand Medical Journal. 2010;123(1311):53-7.

446. Meyer H. Keep it sweet - The role of aspartame in developing healthy and nutritionally balanced foods and drinks. Agro Food Industry Hi-Tech. 2003;14(1):25-7. PubMed PMID: WOS:000182154700007.

447. Pepino MY, Bourne C. Non-nutritive sweeteners, energy balance, and glucose homeostasis. Curr Opin Clin Nutr Metab Care. 2011;14(4):391-5. Epub 2011/04/21. doi: 10.1097/MCO.0b013e3283468e7e. PubMed PMID: 21505330; PubMed Central PMCID: PMC3319034.

448. Popkin BM, Bray GM, Caballero B, Frei B, Willett WC. The role of beverages in a healthy diet: key issues and guidelines. Functional and Speciality Beverage Technology. 2009;(168):451-83. PubMed PMID: WOS:000277265400019.

449. Reyna NY, Cano C, Bermudez VJ, Medina MT, Souki AJ, Ambard M, et al. Sweeteners and beta-glucans improve metabolic and anthropometrics variables in well controlled type 2 diabetic patients. Am J Ther. 2003;10(6):438-43. Epub 2003/11/19. PubMed PMID: 14624282.

450. Rivera JA, Munoz-Hernandez O, Rosas-Peralta M, Aguilar-Salinas CA, Popkin BM, Willett WC. Beverage consumption for a healthy life: recommendations for the Mexican population. Salud Publica De Mexico. 2008;50(2):173-95. doi: Doi 10.1590/S0036-36342008000200011. PubMed PMID: WOS:000255039600010.

451. Schiffman SS, Gatlin CA. Sweeteners: State of knowledge review. Neuroscience and Biobehavioral Reviews. 1993;17(3):313-45.

452. Smeets PAM. Caloric versus low-caloric sweeteners: Can the body be fooled? Edulcorantes calóricos o edulcorantes bajos en calorías?: Es posible engañar al cuerpo? 2010;112(1335):140-53.

453. Stare FJ. Sugar and sugar substitutes in preventive medicine and nutrition. Nutrition and Metabolism. 1975;18(sup.I):133-42.

454. Tandel KR. Sugar substitutes: Health controversy over perceived benefits. Journal of pharmacology & pharmacotherapeutics. 2011;2(4):236-43. Epub 2011/10/26. doi: 10.4103/0976-500X.85936. PubMed PMID: 22025850; PubMed Central PMCID: PMC3198517.

455. Tombek A. Update on sweeteners: Is there anything new about the benefits and risks? Update Süßstoffe Neues über Nutzen und Risiken. 2010;57(4):196-200.

456. Tou JC, Fitch C, Bridges K. SWEETENERS: USES, DIETARY INTAKE AND HEALTH EFFECTS. Bishop MR, editor2010. 1-28 p.

*Letters to the editor, editorials and published conference presentations (n=4)*

457. Blackburn GL. Sweeteners and weight control. World review of nutrition and dietetics. 1999;85:77-87.

458. Chacko E, McDuff I, Jackson R. Replacing sugar-based soft drinks with sugar-free alternatives could slow the progress of the obesity epidemic: Have your Coke® and drink it too. New Zealand Medical Journal. 2003;116(1184).

459. Goran MI. How growing up sweet can turn sour. Pediatric Obesity. 2013;8(4):237-41.

460. Renwick AG, Nordmann H. First European conference on aspartame: Putting safety and benefits into perspective. Synopsis of presentations and conclusions. Food and Chemical Toxicology. 2007;45(7):1308-13. doi: Doi 10.1016/J.Fct.2007.02.019. PubMed PMID: WOS:000247537800028.

*Same review published in different journals (n=1)*

461. Gardner C, Wylie-Rosett J, Gidding SS, Steffen LM, Johnson RK, Reader D, et al. Nonnutritive sweeteners: Current use and health perspectives: A scientific statement from the American heart association and the American diabetes association. Circulation. 2012;126(4):509-19.

*Beverages other than artificially sweetened beverages (n=1)*

462. Pereira MA. The possible role of sugar-sweetened beverages in obesity etiology: a review of the evidence. International Journal of Obesity. 2006;30(SUPPL. 3):S28-S36. doi: Doi 10.1038/Sj.Ijo.0803489. PubMed PMID: WOS:000242337600006.
